# Non-invasive neuromodulation: an emerging intervention for visceral pain in gastrointestinal disorders

**DOI:** 10.1186/s42234-023-00130-5

**Published:** 2023-11-22

**Authors:** Md Jahangir Alam, Jiande D. Z. Chen

**Affiliations:** Division of Gastroenterology and Hepatology, Department of Internal Medicine, University of Michigan, Ann Arbor, MI 48109 USA

**Keywords:** Abdominal pain, Gastrointestinal disorders, Noninvasive neuromodulation, Acupuncture, Transcutaneous electrical acustimulation, Transcutaneous auricular vagus nerve stimulation, Bioelectronic medicine

## Abstract

Gastrointestinal (GI) disorders, which extend from the esophagus to the anus, are the most common diseases of the GI tract. Among these disorders, pain, encompassing both abdominal and visceral pain, is a predominant feature, affecting the patients’ quality of life and imposing a substantial financial burden on society. Pain signals originating from the gut intricately shape brain dynamics. In response, the brain sends appropriate descending signals to respond to pain through neuronal inhibition. However, due to the heterogeneous nature of the disease and its limited pathophysiological understanding, treatment options are minimal and often controversial. Consequently, many patients with GI disorders use complementary and alternative therapies such as neuromodulation to treat visceral pain. Neuromodulation intervenes in the central, peripheral, or autonomic nervous system by alternating or modulating nerve activity using electrical, electromagnetic, chemical, or optogenetic methodologies. Here, we review a few emerging noninvasive neuromodulation approaches with promising potential for alleviating pain associated with functional dyspepsia, gastroparesis, irritable bowel syndrome, inflammatory bowel disease, and non-cardiac chest pain. Moreover, we address critical aspects, including the efficacy, safety, and feasibility of these noninvasive neuromodulation methods, elucidate their mechanisms of action, and outline future research directions. In conclusion, the emerging field of noninvasive neuromodulation appears as a viable alternative therapeutic avenue for effectively managing visceral pain in GI disorders.

**Supplementary Information:**

The online version contains supplementary material available at 10.1186/s42234-023-00130-5.

## Introduction

Pain, including abdominal, visceral, and non-cardiac chest pain, is a common symptom in patients with gastrointestinal (GI) disorders and can be a sign of potential tissue damage, inflammation, or dysfunction of the gut muscles. However, the pathophysiology of the pain associated with these disorders is not entirely understood. Clinical data and research in rodents suggest that sensitization of visceral afferents, spinal dorsal horn, and aberrancies within the descending modulatory systems may play an important role. The brain receives nociceptive signals from the rest of the body and acts on peripheral organs through the nerves innervating these organs. It is assumed that inputs from the overly sensitive nerves of the GI tract muscles and nerves that process these signals in the brain are responsible for pain perception and modulation. This bidirectional crosstalk between the brain and gut ensures proper maintenance of gastrointestinal homeostasis and higher cognitive functions. Therefore, it is imperative to understand the pathophysiology of pain in patients with GI disorders. Previous studies have suggested that brain regions associated with attention (e.g., the anterior cingulate cortex (ACC), medial PFC (mPFC), and thalamus), cognition and emotions (e.g., the hippocampus and amygdala) are involved in pain signal processing in GI disorders. GI disorders are considered bio-psychosocial disorders with unknown etiology; therefore, psychological experiences such as stress, anxiety, and depression could act as mediators in these disorders.

GI disorders, such as functional dyspepsia (FD), irritable bowel syndrome (IBS), and non-cardiac chest pain (NCCP), are commonly observed in patients. Other prevalent GI disorders include gastroparesis and irritable bowel disease (IBD), though to a lesser extent. FD is a gastroduodenal disease (Liu et al. 2008) characterized by epigastric pain or burning, early satiety, and postprandial fullness (Enck et al. 2017; Madisch et al. 2018). Gastroparesis is a chronic motility disorder affecting stomach muscles, interrupting their normal movement and causing delayed emptying of solids in the absence of mechanical obstruction. Symptoms typically include early satiety (60% of patients), postprandial fullness, nausea, vomiting, bloating, and upper abdominal pain (Kim and Kuo 2019; Parkman 2015). IBS, affecting 10% of the global population (Mayer et al. 2023), is characterized by chronic and recurrent abdominal pain with altered bowel habits. Other symptoms of IBS include mucus in the stool, a feeling of incomplete evacuation, visceral sensitivity, and psychiatric conditions such as depression, anxiety, and sleep disturbances. IBD is the chronic inflammation of the digestive tract with symptoms including abdominal pain, diarrhea, rectal bleeding, fatigue, and weight loss. The pathophysiology of IBD may be associated with aberrant or dysregulated immune responses, gut microbial imbalance, intestinal barrier dysfunction, gut motor and sensory dysfunction, and psychological factors (Baumgart and Carding 2007; Song et al. 2019; Strober et al. 2007). Non-cardiac chest pain (NCCP) refers to recurring chest pain that affects approximately 25% of adults in the U.S. (Fass and Navarro-Rodriguez 2008; Jerlock et al. 2007). The underlying mechanisms include gastroesophageal reflux disease (GERD), esophageal hypersensitivity and/or inflammation, IBS, and functional abdominal bloating (Mudipalli et al. 2007).

Functional GI disorders, also known as disorders of gut-brain interaction, are diagnosed using symptom-based criteria; therefore, treating visceral pain in these disorders is challenging. Common treatment methods include antidepressants, such as tricyclic antidepressants (TCAs), selective serotonin reuptake inhibitors (SSRIs), and monoamine uptake inhibitors. A series of studies support the effectiveness of TCAs in decreasing pain perception, having therapeutic effects on mood, sleep, and associated psychological disturbances, and regulating motility (Bahar et al. 2008; Crowell et al. 2004; Grover and Drossman 2008). SSRIs reduce transit time in patients (Gorard et al. 1994), improve overall well-being, reduce anxiety associated with GI-related symptoms, and enhance the analgesic properties of TCAs, suggesting that SSRIs may decrease pain associated with GI disorders. Serotonin-norepinephrine reuptake inhibitors (SNRIs) also help in reducing visceral pain. Histamine (H2) receptor antagonists, such as famotidine, are used for managing symptoms related to delayed gastric emptying in patients with FD. These drugs may also alleviate pain in those patients (See et al. 2001). These drugs reduce visceral and neuropathic pain by acting on the central and peripheral nervous systems (Lebel 2008; Whitfield and Shulman 2009). Given the heterogeneous pathophysiology of GI disorders, the treatment of visceral pain remains a considerable therapeutic challenge; therefore, alternative treatment methods, such as neuromodulation, could be beneficial.

The aim of this article is to provide a systematic review of the therapeutic potential of noninvasive neuromodulation for visceral pain in a few major functional GI diseases and IBD.

## Neuromodulation

Neuromodulation refers to the interfacing and intervention in the central, peripheral, or autonomic nervous systems. Neuromodulation acts directly on nerves by alternating or modulating nerve activity using electrical, electromagnetic, chemical, or optogenetic methods (Krames et al. 2009). The modern era of neuromodulation began in the early 1960s with deep brain stimulation (DBS), followed by spinal cord stimulation in 1967, used for the treatment of chronic and intractable pain. Spinal cord stimulation (SCS) is a well-established method for treating chronic pain, particularly neuropathic pain. It is also employed in the treatment of ischemic pain, such as angina and chronic critical limb ischemia, visceral pain associated with chronic pancreatitis, and pelvic pain disorders. Given the broad therapeutic scope of neuromodulation and its ongoing improvements, it can be theorized that this approach could be used to treat a wide and diverse range of conditions, including the management of visceral pain in GI disorders. Considering its applications, neuromodulation can be categorized into two types.

### Invasive neuromodulation

Invasive neuromodulation refers to a technique that requires the placement of stimulating electrodes through surgical intervention in the vicinity of the neural substrate. Several invasive neuromodulation methods have received approval from the US Food and Drug Administration (FDA) for the treatment of neurological disorders. For example, vagus nerve stimulation (VNS) is used for treating epilepsy and depression (Afra et al. 2021), while deep brain stimulation (DBS) is employed for obsessive-compulsive disorder (Bergfeld et al. 2021) and movement disorders, including Parkinson’s disease (Bari et al. 2018; Weaver et al. 2009). Other methods, such as SCS, are used to alleviate neuropathic pain, and sacral nerve stimulation (SNS) is applied in the treatment of pelvic disorders and incontinence.

### Noninvasive neuromodulation

Noninvasive neuromodulation can be defined as a method that can penetrate the skin to stimulate nerves by delivering electrical stimuli. The most studied noninvasive neuromodulation methods include electroconvulsive therapy (ECT), transcranial electrical stimulation (TES), transcranial magnetic stimulation (TMS), transcranial direct current stimulation (tDCS), and transcranial alternating current stimulation (tACS). Repetitive transcranial magnetic stimulation (rTMS) has been used to treat medication-resistant major depression. Other noninvasive neuromodulation techniques include acupuncture, electroacupuncture (EA), transcutaneous auricular vagal nerve stimulation (taVNS), transcutaneous vagal nerve stimulation (tVNS), transcutaneous electrical acustimulation (TEA) or transcutaneous electrical acupoint stimulation (TEAS), translumbosacral anorectal magnetic stimulation (TAMS), and transcutaneous tibial nerve stimulation (tTNS).

1. Acupuncture and electroacupuncture: Acupuncture is a complex therapeutic method that can be achieved by stimulating different points (acupoints) necessary for normal body function (White and Ernst 2004; Yin and Chen 2010). These acupoints can be stimulated manually or electrically. In the conventional approach, a thin stainless-steel needle is inserted into an acupoint and manually manipulated in various ways, including thrusting, lifting, twisting, twirling, or a combination of these techniques. Alternatively, the inserted needle can be stimulated by electric pulses known as electroacupuncture (EA). Both types of acupuncture have become popular worldwide for the treatment of various disorders, including Alzheimer’s disease (Jiang et al. 2021; Wu et al. 2023), migraines (Diener et al. 2006; Linde et al. 2005), and pain (Cheng and Pomeranz 1979; Pomeranz and Warma 1988). The effects of acupuncture and EA in managing pain and other symptoms of GI disorders have been extensively studied. For instance, stimulation of the stomach acupoint (ST36) has shown improvement in multiple GI symptoms, such as abdominal pain, diarrhea, constipation, gas, bloating, and nausea (Song et al. 2019). Several studies have demonstrated the effectiveness of acupuncture in managing IBD, both in human clinical trials and experimental models of IBD (Bao et al. 2022, 2014; Jin et al. 2017). Acupuncture exerts analgesic effects on visceral pain through various mechanisms, including signal interaction and integration at neurons, ion channels, activation of central descending inhibitory pathways, gene regulation, and the recruitment of various biochemicals, such as opioids, 5-HT, and NMDA (Zhang et al. 2014b; Zhao 2008).
2. taVNS or aVNS: The vagus nerve comprises 80% sensory afferent fibers and only 20% motor efferent fibers. The vagal nerve fibers innervate peripheral organs such as the esophagus, lungs, liver, stomach, and intestines (Bonaz et al. 2013; Liu et al. 2020a) and regulate the function of the autonomic nervous system, whereas the brain receives information from these organs through afferent projections of the vagus and relays visceral, somatic, and taste sensations (Yu et al. 2008). Therefore, the vagus nerve serves as a bidirectional communication pathway between the brain and the body. The afferent fibers of the vagus directly project to the nucleus tractus solitarius (NTS) (Cooper et al. 2021) and form direct and indirect ascending projections from the NTS to many areas of the brain, including the locus coeruleus (LC), midbrain, hypothalamus, amygdala, hippocampus, and frontal lobe (Alam and Chen 2023; Chen et al. 2023; Lopes et al. 2016). taVNS is a new, non-invasive neuromodulation method that targets the auricular branch of the vagus nerve (Farmer et al. 2020; Redgrave et al. 2018). This method offers broader applicability due to its non-invasiveness. taVNS has shown promise in the treatment of various neurological disorders, including epilepsy and depression (Bauer et al. 2016; Hein et al. 2013), tinnitus (Lehtimaki et al. 2013), chronic pain (Napadow et al. 2012), and modulation of attention and cognition (Fischer et al. 2018). Furthermore, taVNS has demonstrated positive results in managing abdominal pain and constipation in patients with IBS (Mion et al. 2020; Shi et al. 2021) and opioid-induced constipation in rats (Zhang et al. 2021c). A recent study also supports the effectiveness of taVNS in the treatment of FD (Shi et al. 2023), while another study suggests that taVNS can attenuate IBD in children (Sahn et al. 2023). These findings emphasize the potential of taVNS for managing visceral pain in GI disorders. Owing to its non-invasiveness, cost-effectiveness, ease of use, and efficacy, taVNS is an appealing neuromodulation method for the treatment of a wide range of disorders (Yap et al. 2020).
3. TEA or TEAS: TEA or TEAS is a noninvasive electrical neuromodulation technique that uses low-frequency pulse current to stimulate selected acupoints through surface electrodes (Francis and Johnson 2011; Mahmood et al. 2019; Wang et al. 2023). Fundamentally, TEAS combines transcutaneous electrical nerve stimulation techniques with acupuncture, offering a dual effect. This method can be applied to several acupuncture points, including the most commonly used ST36 (Zusanli, below the kneecap, in the vicinity of the peroneal, tibial, and sciatic nerves) and PC6 (Nanguan point on the wrist over the medial nerve). TEAS is widely used in clinical practice because of its simplicity, non-invasiveness, painlessness, and other characteristics. Previous studies revealed the beneficial effects of TEAS in reducing episodes of nausea and vomiting (Chen et al. 2020), promoting the recovery of gastrointestinal function (Li et al. 2021), and improving postoperative pain (Parseliunas et al. 2021; Tu et al. 2019; Zhang et al. 2018a). Other research studies have demonstrated the ameliorating effects of TEAS on GI disorders, including GERD, FD, IBS, and constipation (Ji et al. 2014; Xiao et al. 2022; Zhang et al. 2021a). Moreover, TEAS can accelerate postoperative gastrointestinal function recovery, reduce postoperative hospital stay, and improve daily activities in patients undergoing cesarean section and thoracoscopic surgery (Yang et al. 2020; Zhou et al. 2018).

## Methods of systematic review

We conducted a search of databases, including PubMed, Web of Science, and Scopus, and included studies published only in English without restricting the year of publication. We used the following inclusion and exclusion criteria for screening: included studies involving human patients. Moreover, we only included randomized controlled trials (RCTs) on acupuncture and electroacupuncture. We excluded studies conducted on animals, systematic reviews and meta-analyses, patients in mechanically ventilated or ICU, and non-empirical studies, such as editorial letters, conference proceedings, meeting abstracts, commentary, and authors’ replies.

For acupuncture and EA, we used the following keywords to search the literature: (“acupuncture”) OR (“electroacupuncture”) AND (gastroparesis) OR (“Functional dyspepsia”) OR (“delayed gastric emptying”) OR (“gastric motility”) OR (“noncardiac chest pain”) OR (“irritable bowel syndrome”) OR (IBS) OR (“Inflammatory bowel disease”) OR (IBD)). We retrieved 3129 articles from PubMed (*n* = 1298), Scopus (*n* = 884), and the Web of Science (*n* = 947). Duplicate articles were removed by filtering through the title column using an automated tool (Python. Subsequently, three filtering criteria were applied in Python (Supplementary method). We applied three additional filters (search by condition, dropping keywords, and final keywords) and retrieved 242 articles using an automated screening method, which were manually checked based on the inclusion criteria. We found 20 articles that matched our inclusion criteria (Tables 1 and 2).

**Table 1:** The effectiveness of acupuncture in treating visceral pain in GI disorders

Articles	Acupoints	Stimulation parameter	Number of participants	Disease treated	Study design	Main findings	Pain specific
(Pei et al. 2020)	GV20, GV29, LR3, ST36, SP6, ST25, and ST37	Three times a week for 6 weeks. The needles were manipulated every 10 min, and each session lasted 30 min.	AP (*n* = 344) PEG 4000/pinaverium bromide (*n* = 175)	IBS-C and IBS-D	Multicenter RCT	AP treatment significantly improved total IBS-SSS scores (123.51 vs 94.73, *P* < 0.01), including the severity of abdominal pain (20.92 ± 25.41 vs 13.43 ± 21.48, *P* = 0.003) when compared with PEG 4000/pinaverium bromide group. Furthermore, AP treatment improved IBS-QOL total scores (13.35 vs 8.95, *P* = 0.02).	Yes
(Shen et al. 2022)	ST25, CV12, ST36, ST37, SP4, ST40, LR13, and SP9	Three times a week for 8 weeks. Each session lasted 30 min, and the needles were manipulated every 10 min.	AP (*n* = 33) Sham-AP (*n* = 32)	IBS-D	RCT	Both groups of AP and Sham-AP had reduced IBS-SSS scores, including pain, compared with the baseline. However, there was a significant difference in the IBS-SSS score during the follow-up visit between the groups (169.70 ± 54.11 vs 204.38 ± 52.48, *P* < 0.05). Moreover, the BSS score also decreased after the treatment (5.00 ± 0.83 vs 5.44 ± 0.72, *P* < 0.05) and during follow-up (5.06 ± 0.86 vs 5.53 ± 0.84, *P* < 0.05) in the AP group comparing to the Sham-AP group.	Yes
(Rafiei et al. 2014)	UB17, 23, 25 DU3, SP9, 15, ST25, 36, Ren12, and 4. Kid15	Catgut AP method	AP (*n* = 20) Sham-AP (*n* = 20) DO (*n* = 20)	IBS	RCT Double-blinded	AP treatment decreased abdominal pain when compared with the Sham-AP and DO group (mean score, AP: 3.07, Sham-AP: 4.6, DO: 5.08, *P* = 0.003). AP treatment also decreased symptoms of depression when compared with the DO group (mean score, AP: 15.9, DO: 21.9, *P* = 0.002).	Yes
(Lowe et al. 2017)	ST25, ST34, ST36, ST37, UB20 and UB23, LI4, CV6, and CV12.	Treatment was given twice weekly for 4 weeks, and the session lasted 30 min.	AP (*n* = 43) Sham-AP (*n* = 36)	IBS	RCT	This study didn’t observe significant differences between groups in abdominal pain score (51.0 ± 27.1 vs 38.6 ± 17.2, *P* = 0.70) and in McGill pain score (2.6 ± 0.4 vs 3.5 ± 0.5, *P* = 0.19), though both groups demonstrated reduction from the baseline. Furthermore, similar results were observed in IBS symptoms score (12.2 ± 1.2 vs 13.9 ± 1.6, *P* = 0.40) and QOL measures (2.2 ± 0.2 vs 1.9 ± 0.2, *P* = 0.28).	Yes
(Qi et al. 2022)		Three sessions per week, every other day for 4 weeks, each session lasting 30 min.	SA (*n* = 30) NSA (*n* = 30) NA (*n* = 30)	IBS-D	Multicenter RCT	Acupuncture in both SA and NSA groups improved abdominal pain and IBS-SSS scores, although there were no significant differences among the three groups.	Yes
(Zhang et al. 2019a)	CV4, PC6, ST36, SP6, ST25, and ST37.	Three (3) sessions per week, every other day, for 4 weeks, each session lasting 30 min.	AP (*n* = 31) Control (*n* = 30)	IBS-D	RCT	AP treatment significantly decreased IBS-SSS scores, including abdominal pain (100.97 ± 8.55 vs 254.17 ± 11.98, *P* < 0.05), compared to the control group. AP treatment also improved anxiety (7.06 ± 0.50 vs 9.87 ± 0.51, *P* < 0.05) and depression (6.29 ± 0.42 vs 10.43 ± 0.49, *P* < 0.05).	Yes
(Meng 2019)	LR3, ST36, ST37, SP6, ST25, GV20, and GV29.	Five times a week for 4 weeks, and each session lasted for 30 min	AP (*n* = 35) Control (*n* = 35)	IBS-D	RCT	AP treatment decreased the total IBS-SSS score (193.71 ± 52.42 vs 245.14 ± 47.36, *P* < 0.05), including the degree of abdominal pain (36.00 ± 26.48 vs 48.57 ± 25.80, *P* < 0.05), frequency of abdominal pain (30.29 ± 27.17 vs 50.29 ± 23.45, *P* < 0.05), and defecation satisfaction (38.29 ± 29.25 vs 50.86 ± 22.93, *P* < 0.05) compared to the control group. AP treatment also decreased their depression state (51.17 ± 12.92 vs 60.69 ± 16.26, *P* < 0.05).	Yes
(Li et al. 2015)	ST36, PC6, and CV12	Once a day for 7 days, each session lasted for 20–30 min	AP (*n* = 11) Sham-AP (*n* = 10)	GP	Single-blind, crossover trial	Although both groups had reduced GSCI and GVAS scores from the baseline, the AP group showed significant differences in both GSCI (− 8.0 ± 3.4 vs − 2.4 ± 3.7, *P* < 0.01) and GVAS (− 58.1 ± 31.2 vs − 12.9 ± 29.9, *P* < 0.01) scores when compared with the sham group.	Yes
(Jin et al. 2015)	ST36, KI3, GB4, PC6, and HT7	Three sessions per week, every other day, for 4 weeks	Treatment (*n* = 30) Control (*n* = 30)	FD	RCT	DSS scores, including epigastric pain, were reduced in both groups; however, the treatment group exhibited more effect than the control group (0.48 ± 1.03 vs 6.32 ± 3.41, *P* < 0.0001). AP treatment has better outcomes for improving mental status [(SDS score (45.60 ± 8.75 vs 54.00 ± 10.80, *P* < 0.0001)) and SAS score (42.30 ± 6.22 vs 52.20 ± 7.98, *P* < 0.0001))] and QOL [SF-36 score (70.0 ± 12.54 vs 56.00 ± 13.42, *P* < 0.0001)].	Yes
(Lee et al. 2022)	Saam acupuncture	Three sessions per week for 4 weeks, and each session lasted 20 min.	Saam-AP (*n* = 12) Usual care (*n* = 12)	FD	RCT	There was no difference in GIS after the treatment between both groups. However, Saam-AP had a significant reduction in GIS scores at 8-week [(6.30 (3.03, 9.57) vs 9.40 (5.64, 13.16), *P* = 0.0339] and 12-week [(4.70 (1.26, 8.14) vs 10.80 (5.22, 16.38), *P* = 0.0113] follow-ups compared to the usual care group. Only Saam-AP group significantly reduced epigastric pain scores at 8-week [(2.10 (1.24, 2.96) vs 0.70 (0.11, 1.29), *P* = 0.0205] and 12-week follow-ups [[(2.10 (1.24, 2.96) vs 0.60 (0.10, 1.10), *P* = 0.0091] when compared to the baseline.	Yes
(Xuefen et al. 2020)	Group-A: CV12, ST36 Group-B: PC6, ST36 Group-C: non-acupoints, ST36	Five sessions per week for 3 weeks, and each session lasted 30 minutes.	Group-A (*n* = 33) Group-B (*n* = 33) Group -C (*n* = 33)	GP	RCT	All groups demonstrated a significant reduction in GCSI scores. However, Group-A had better outcomes than all groups.	No
(Ko et al. 2016)	LI4, ST36, LR3, SP4, CV12, GB21, SI14, PC6, EX-HN5, and ST34	Twice weekly for 4 weeks	AT (*n* = 37) Control (*n* = 39)	FD	RCT	Acupuncture treatment significantly reduced the total NDI score (57.1 ± 30.2 43.4 ± 33.1, *P* = 0.03) compared to the control group. Moreover, AP treatment significantly reduced upper abdominal pain (4.3 ± 3.6 vs 3.2 ± 3.6, *P* < 0.05) and discomfort in the upper abdomen (6.8 ± 3.4 4.7 ± 3.8, *P* < 0.05) from the baseline; however, there was a significant difference between groups in discomfort in the upper abdomen (6.4 ± 3.2 4.7 ± 3.8, *P* = 0.01).	Yes
(Park et al. 2009)	CV12, LI4, LR3, ST36, PC6, and SP4.	Three times per week for 2 weeks After insertion, needles were rotated 90° for 3 times and retained for 15 min.	AP (*n* = 38) Sham-AP (*n* = 38)	FD	RCT	In both cases, NDI scores, including abdominal pain, reduced from the baseline (from 59.59 ± 22.03 to 25.44 ± 17.96, *P* < 0.001 in the AP group, and from 55.71 ± 22.94 to 26.38 ± 15.69, *P* < 0.001 in the Sham-AP group). However, no significant differences were observed in the average NDI scores between groups (34.15 ± 24.74 vs 29.32 ± 20.76, *P* = 0.387).	Yes

*Abbreviations*: *AP* Acupuncture, *Sham-AP* Sham acupuncture, *AT* Actual treatment, *NDI* The Nepean dyspepsia index, *GV20* Baihui, *GV29* Yintang, *LR3* Taichong, *ST36* Zusanli, *SP6* Sanyinjiao, *ST25* Tianshu, *ST37* Shangjuxu, *CV12* Zhongwan, *SP4* Gongsun, *ST40* Fenglong, *LR13* Zhangmen, *SP9* Yinlingquan, *PC6* Neiguan, *BL25* Dachangshu, *LI11* Quchi, *GB40* Qiuxu, *GB37* Guangming, *GB34* Yanglingquan, *GB36* Waiqiu, *ST33* Yinshi, *ST35* Dubi, *ST32* Futu, *ST34* Liangqiu, *ST42* Chongyang, *ST38* Tiaokou, *BL21* Weishu, *KI3* Yuan, *ST2* Neiting, *SP4* Gongsun, *SP9* Yinlingquan, *Ren4* Guanyuan, *Ren12* Zhongwan, *Kid15* Zhongzhu, *EX-HN5* Taiyang, *HT7* Shenmen, *DU3* Yaoyangguan *LI-EA* Low intensity EA, *HI-EA* High intensity EA, *IBS-C* Irritable bowel syndrome with constipation, *IBS-D* Irritable bowel syndrome with diarrhea, *FD* Functional dyspepsia, *GP* Gastroparesis, *RCT* Randomized controlled trial, *SA* Specific acupoints, *NSA* Non-specific acupoints, *NA* Non-acupoints, *IBS-SSS* Irritable Bowel Syndrome Severity Scoring System, *IBS-QOL* Irritable bowel syndrome quality of life, *GCSI* Gastroparesis cardinal symptom index

**Table 2:** Electroacupuncture in treating visceral pain in GI disorders

Articles	Acupoints	Stimulation parameter	Number of participants	Disease treated	Study design	Main findings	Pain specific
(Zheng et al. 2016)	LI11 and ST37 or ST25 and BL25 or LI11, ST37, ST25 and BL25.	15 Hz continuous wave for 30 min total of 16 sessions over 4 weeks	He-EA (*n* = 113) Shu-Mu-EA (*n* = 111) He-Shu-Mu-EA (*n* = 112) Loperamide (*n* = 112)	IBS-D	RCT	All groups had significantly reduced stool frequency from baseline. However, no differences were observed among the groups.	No
(Xu et al. 2022b)	ST25 and BL25	LI-EA: 2/50 Hz, 0.1–0.8 mA HI-EA: 2/50 Hz, 1.0–1.8 mA	LI-EA (*n* = 25) HI-EA (*n* = 26) Loperamide (*n* = 22)	Functional Diarrhea	RCT	EA, especially LI-EA, significantly improved the proportion of normal defecation. EA also improved stool consistency and weekly spontaneous bowel movements. LI-EA also effectively improved QOL, anxiety, and depression, suggesting that LI-EA may have better outcomes when compared with the loperamide group.	No
(Ma et al. 2012)	Group-A: ST34, 36, 40, 42 Group-B: ST32, 33, 35, 38 Group-C: BL21, CV12 Group-D: GB34, 36, 37, 40 Group-E: Non-acupoints Group-F: Itopride	2/100 Hz, 0.5–1.5 mA, 5 consecutive days in a week for 4 weeks, and each session lasted for 30 min.	Group-A (*n* = 118) Group-B (*n* = 120) Group-C (*n* = 116) Group-D (*n* = 119) Group-E (*n* = 120) Group-F (*n* = 119)	FD	RCT	All groups had improved SID scores, including epigastric pain, from baseline; however, AP groups (A, B, C, and D) and group-F were superior to the Sham-AP group (all *P* < 0.05 vs. Sham-AP). Group-A had significant differences in SID scores (2.43 ± 1.88 vs. 1.76 ± 2.24, *P* = 0.005) and QOL scores (14.8 ± 11.8 vs 8.23 ± 11.6, *P* < 0.001) when compared with group-F.	Yes
(Zheng et al. 2018b)	ST36, PC6, TR3, ST2, SP4, ST36, and SP9	2/100 Hz, 0.1–1.0 mA for 30 min, 5 days a week for 4 weeks	EA (*n* = 100) Sham-EA (*n* = 100)	FD	RCT	Both groups had reduced LDQ scores, including epigastric pain, from the baseline (EA: 7.65 ± 3.8 to 2.62 ± 2.60; Sham-EA: 6.86 ± 3.00 to 5.06 ± 3.13). However, the EA group was superior to Sham-EA (mean difference, –2.2, *P* < 0.001). Furthermore, the effect lasted for at least 20 weeks in the EA group	Yes
(Wu et al. 2017)	LI11 and ST37	Five times a week for 2 weeks, then 3 times per week for another 2 weeks LI-EA: 2/50 Hz, low intensity HI-EA: 2/50 Hz with high intensity.	LI-EA (*n* = 62) HI-EA (*n* = 68) Mosapride (*n* = 71)	Functional constipation	RCT	All treatments improved SBM scores and reduced straining severity. HI-EA also improved the QOL better than mosapride.	No
(Zheng et al. 2018a)	LI11 and ST37 or ST25 and BL25 or LI11, ST37, ST25 and BL25	15 Hz continuous wave, 5 times a week for 2 weeks, then 3 times per week for another 2 weeks	He-EA (*n* = 172) Shu-Mu-EA (*n* = 168) He-Shu-Mu-EA (*n* = 165) Mosapride (*n* = 170)	Functional constipation	RCT	The spontaneous bowel movement increased in all groups without any significant difference among groups, suggesting that EA is as effective as mosapride.	No
(Liu et al. 2020b)	ST36, PC6, LR3, ST44, SP4, and SP9	2 Hz/100 Hz, 0.1–1.0 mA based on the patient’s tolerance, and the patient was stimulated for 30 min.	EA (*n* = 33) Sham-EA (*n* = 35)	FD	RCT	EA treatment significantly reduced upper abdominal pain (0.82 ± 1.01 vs 1.43 ± 1.07, *P* < 0.05), postprandial satiety (1.15 ± 1.09 vs 1.97 ± 0.95, *P* < 0.01), and upper abdominal burning sensation (0.18 ± 0.47 vs 0.77 ± 1.09, *P* < 0.01) compared to the control group.	Yes

*Abbreviations*: *EA* Electroacupuncture, *Sham-EA* Sham elelctroacupuncture, *LDQ* Leeds Dyspepsia Questionnaire, *SID* Symptom Index of Dyspepsia, *BL21* Weishu, *BL25* Dachangshu, *CV12* Zhongwan, *LI11* Quchi, *PC6* Neiguan, *SP4* Gongsun, *SP6* Sanyinjiao, *SP9* Yinlingquan, *ST2* Neiting, *ST25* Tianshu, *ST32* Futu, *ST33* Yinshi, *ST34* Liangqiu, *ST35* Dubi, *ST36* Zusanli, *ST37* Shangjuxu, *ST38* Tiaokou, *ST40* Fenglong, *ST42* Chongyang, *TR3* Taichong, *GB40* Qiuxu, *GB37* Guangming, *GB34* Yanglingquan, *GB36* Waiqiu, *LI-EA* Low intensity EA, *HI-EA* High intensity EA, *SBM* Spontaneous bowel movement, *QOL* Quality of life, *IBS-D* Irritable bowel syndrome with diarrhea, *FD* Functional dyspepsia, *RCT* Randomized controlled trial

For transcutaneous auricular vagus nerve stimulation (taVNS) and auricular vagus nerve stimulation (aVNS), the following combination of keywords was used for literature searching: (“auricular vagus nerve stimulation”) OR (“auricular vagus nerve stimulation”) OR (“transcutaneous auricular vagus nerve stimulation”) OR (“transcutaneous auricular vagal nerve stimulation”) OR (taVNS) OR (aVNS) AND (gastroparesis) OR (“Functional dyspepsia”) OR (“delayed gastric emptying”) OR (“gastric motility”) OR (“noncardiac chest pain”) OR (“irritable bowel syndrome”) OR (IBS) OR (“Inflammatory bowel disease”) OR (IBD)). We retrieved 1057 articles from PubMed (*n* = 1019), Scopus (*n* = 19), and Web of Science (*n* = 19). We then applied the same prescreening method as mentioned earlier and retrieved eight articles, reviewed abstracts, and full texts. Four studies matched the requirements and were included in the report (Table 3). Furthermore, percutaneous electrical nerve field stimulation (PENFS) is another noninvasive neuromodulation method approved by the FDA for the treatment of abdominal pain. While this method does not explicitly target the vagus nerve, both taVNS and PENFS may share the same pain signaling pathway. Consequently, we have included two additional references in Table 3.

**Table 3:** Effect of taVNS for the management of pain in GI disorders

Articles	Stimulation parameter	Number of participants	Disease treated	Study design	Main findings	Pain specific
(Steidel et al. 2021)	1 Hz and 25 Hz (250 µs, 30 s ON/30 s OFF) for 4 h	taVNS-1 Hz (*n* = 28) taVNS-25 Hz (*n* = 24)	Healthy	Randomized, double-blinded	This study demonstrated that high frequency taVNS influenced gastric motility through higher amplitudes of peristaltic waves in the antrum.	No
(Zhu et al. 2021)	25 Hz, 0.5 ms pulse width, 2 s ON, 3 s OFF, pulse amplitude of 0.5 mA to 1.5 mA 1 h, twice daily for 2 weeks	taVNS (*n* = 18) Sham-ES (*n* = 18)	FD	Randomized, double-blinded	taVNS increased gastric accommodation (901.2 ± 39.6 mL vs. 797.1 ± 40.3 mL, *P* < 0.001), reduced the scores of dyspeptic symptoms, including pain [2.0 (0.0, 2.0) vs. 2.0 (2.0, 4.0), *P* = 0.046, *n* = 6], and reduced both anxiety [5.5 (1.0, 14.0) vs. 8.0 (4.0, 16.0), *P* = 0.002] and depression scores [2.5 (0.0, 8.0) vs. 5.0 (1.0, 12.0), *P* < 0.001] when compared with the baseline.	Yes
(Wu et al. 2021)	30 Hz, continuous wave, 5 times a week, 30 min each time, for 4 weeks	taVNS (*n* = 45) tnVNS (*n* = 45)	FD	Randomized, double-blinded	taVNS improved the overall symptom points, including upper abdominal pain (10.27 ± 3.43 vs 15.29 ± 2.95, *P* < 0.05), FDQOL (60.99 ± 3.25 vs 58.43 ± 4.63, *P* < 0.05), and reduced the score of anxiety (13.51 ± 5.16 vs 15.82 ± 4.38, *P* < 0.05) and depression (12.36 ± 3.67 vs 14.18 ± 3.14, *P* < 0.05) compared with tnVNS group.	Yes
(Shi et al. 2021)	25 Hz, 0.5 ms pulse width, 2 s ON, 3 s OFF, 1 h for 4 weeks, each session lasted for 30 min.	taVNS (*n* = 21) Sham-taVNS (*n* = 21)	IBS-C	Randomized	taVNS reduced the VAS pain score (3.1 ± 2.2 vs 1.1 ± 1.1, *P* = 0.001), improved constipation (0.9 ± 0.9 vs 2.8 ± 2.2, *P* = 0.001), QOL (69.5 ± 21.2 vs 83.2 ± 12.5, *P* = 0.020) when compared with the sham-taVNS. Furthermore, taVNS decreased pro-inflammatory cytokines, including TNF-α (6.7 ± 3.0 pg/mL vs 3.9 ± 2.1 pg/mL, *P* = 0.001) and IL-6 (3.4 ± 2.8 pg/mL vs 1.9 ± 1.1 pg/mL, *P* = 0.037), and plasma-5HT (50.0 ± 15.4 ng/mL vs 38.5 ± 15.4 ng/mL, *P* = 0.007) when compared with baseline.	Yes
(Krasaelap et al. 2020)	One and 10 Hz, 1-ms pulse, every 2 s, continuously cycling 2 h on and 2 h off for a total of 120 h (5 days), —five days per week for a total of 4 weeks.	PENFS (*n* = 27) Sham (*n* = 23)	IBS	Randomized, double-blinded trial	Thirty percent reduction of worst pain score was observed in 59% of patients who received PENFS stimulation vs 26% of patients who received the sham stimulation (*P* = .024). Moreover, PENFS reduced composite pain score (7.5 vs 14.4, *P* = .026) and usual pain score (3.0 vs 5.0, *P* = .029) compared with sham stimulation.	Yes
(Kovacic et al. 2017)	One and 10 Hz, 1-ms pulse, every 2 s, continuously cycling 2 h on and 2 h off for a total of 120 h (5 days). Five days per week for a total of 4 weeks.	PENFS (*n* = 57) Sham (*n* = 47)	Abdominal pain-related FGIDs	Randomized, double-blind, sham-controlled trial	PENFS stimulation improved both worst pain score (5.0 vs 7.0, *P* = 0.003) and composite pain score (8.4 vs 15.2, *P* < 0.0001) in patients compared with sham stimulation.	Yes

*Abbreviations*: *tnVNS* Transcutaneous non-vagus nerve stimulation, *QOL* Quality of life, *PENFS* Percutaneous electrical nerve field stimulation, *taVNS* Transcutaneous auricular vagus nerve stimulation, *IBS-C* Irritable bowel syndrome with constipation, *FD* Functional dyspepsia, *QOL* Quality of life, *FDQOL* Functional dyspepsia quality of life

**Fig. 1:**
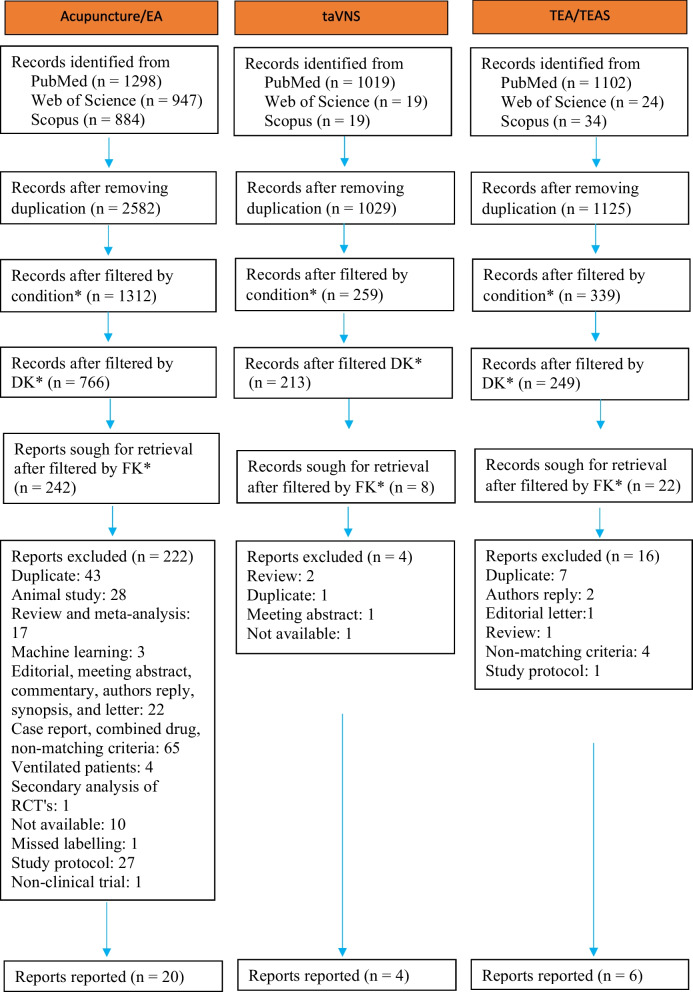
PRISMA flow diagram. Note: Following the screening and reviewing process guidelines based on Page et al. (2021). Abbreviations: DK, Dropping keywords; FK, Final keywords

For transcutaneous electrical stimulation (TEA) and transcutaneous electrical acustimulation (TEAS), we found a total of 1160 articles from PubMed (*n* = 1102), Scopus (*n* = 24), and Web of Science (*n* = 34) with the following search terms: (“transcutaneous electrical stimulation”) OR (“transcutaneous electrical acustimulation”) OR (“transcutaneous electrical acu-stimulation”) AND (gastroparesis) OR (“Functional dyspepsia”) OR (“delayed gastric emptying”) OR (“gastric motility”) OR (“noncardiac chest pain”) OR (“irritable bowel syndrome”) OR (IBS) OR (“Inflammatory bowel disease”) OR (IBD)). Articles were filtered using an approach similar to that described in this review. The number of reports was adjusted to 22, the abstracts and full texts were, and nine papers matched the requirements and were included for reporting (Table 4). The literature search method is summarized below (Fig. 1).

**Table 4:** TEAS for treating visceral pain in GI disorders

Articles	Acupoints	Stimulation parameter	Number of participants	Disease treated	Study design	Main findings	Pain specific
(Xing et al. 2004)	ST36, P6	5 Hz, 250 ms	*n* = 7	IBS-D	No randomization	Stimulation at ST36 and P6 improved VAS pain score at different pressure [8 mmHg (176.2 ± 19.9 vs 221.1 ± 30.0, *P* < 0.01), 16 mmHg (209.3 ± 21.15 vs 252.0 ± 40.6, *P* < 0.01), and 32 mmHg (237.8 ± 23.7 vs 248.4 ± 31.0, *P* < 0.01)] compared to the sham stimulation.	Yes
(Song et al. 2018)	PC6, ST36	25 Hz, 2 s ON, 3 s OFF, amplitude (2–10 mA) 1 h	*n* = 18	GP	Randomized, Placebo-controlled	TEA improved gastric dysrhythmia.	No
(Huang et al. 2022)	ST36, PC6	25 Hz, 0.5 ms pulse width, 2 s ON, 3 s OFF, amplitude (2–10 mA) twice daily for 4 weeks, and each session was for 1 h	TEA (*n* = 26) Sham-TEA (*n* = 26)	IBS-C	Randomized, Single-blind	TEA reduced VAS pain score (1.34 ± 1.1 vs 2.32 ± 1.0, *P* = 0.002), improved constipation, and IBS-QOL (86.4 ± 9.5 vs 76.5 ± 12.8, *P* = 0.004) than Sham-TEA. The effect of TEA lasted at least 6 months. Furthermore, TEA enhanced vagal activity, accelerated colon transit (71.6% ± 15.7% vs 47.92% ± 31.3, *P* = 0.002), and increased the threshold of rectal sensation (85.0 ± 16.8 vs 73.3 ± 8.2, *P* = 0.004) than Sham-TEA.	Yes
(Hu et al. 2022)	ST36, LI4	ST36: 25 Hz, 0.5 ms pulse width, 2 s ON, 3 s OFF LI4: 100 Hz, 0.1 s ON, 0.4 s OFF, 0.5 ms pulse width 30 min, twice daily for 1 month	TEA (*n* = 21) Sham-TEA (*n* = 16)	IBS-D	Randomized	TEA reduced abdominal pain (VAS score), which was higher than the sham-TEA group (3.5 ± 2.00 vs 1.0 ± 1.88, *P* = 0.014) and improved IBS-QOL before the treatment (78.55 ± 9.62 vs 85.97 ± 9.49, *P* < 0.0001).	Yes
(Hu et al. 2020)	ST36, PC6	25 Hz, 0.5 ms pulse width, 2 s ON, 3 s OFF, 30 min	(*n* = 30)	GERD	Randomized, Acute	TEA increased gastric accommodation, improved gastric slow waves, and reduced postprandial fullness, suggesting that TEA may improve NCCP in GERD.	No
(Zhang et al. 2021a)	ST36, PC6	25 Hz, 0.5 ms pulse width, 2 s ON, 3 s OFF, 30 min, twice daily for 1 month	TEA (*n* = 15) Sham-TEA (*n* = 15)	GERD	Randomized	TEA improved GERDQ scores, including upper stomach pain (7.1 ± 1.2 vs 9.3 ± 2.7, *P* = 0.011) and GERD-HRQL scores (3.1 ± 3.8 vs 7.2 ± 5.9, *P* = 0.028) than Sham-TEA group.	Yes

*Abbreviations*: *ST36* Zusanli, *PC6* Neiguan, *LI4* Hegu, *GERD* Gastroesophageal reflux disease, *GP* Gastroparesis, *IBS-C* Irritable bowel syndrome with constipation, *IBS-D* Irritable bowel syndrome with diarrhea, *TEA* Transcutaneous electroacupuncture, *NCCP* Noncardiac chest pain, *GERDQ* Gastroesophageal reflux disease questionnnaire

## Results

### Acupuncture/electroacupuncture

We retrieved 242 articles using an automated screening method, which was manually checked based on the inclusion criteria. We found 20 articles that matched our inclusion criteria, as reported in Tables 1 and 2. We extracted the following outcomes: IBS symptom severity scale (IBS-SSS) (Meng 2019; Pei et al. 2020; Qi et al. 2022; Shen et al. 2022; Zhang et al. 2019a); Gastroparesis cardinal symptom index (GCSI) (Li et al. 2015; Xuefen et al. 2020); Gastroparesis visual analog pain (Li et al. 2015); Dyspeptic symptom score (DSS) (Jin et al. 2015); Gastrointestinal symptoms score (GIS) (Lee et al. 2022); Visual analog pain scores (VAS) (Lee et al. 2022; Rafiei et al. 2014); Nepean Dyspepsia Index (NDI) (Ko et al. 2016; Liu et al. 2020b; Ma et al. 2012; Park et al. 2009); Leeds Dyspepsia Questionnaire (LDQ) (Liu et al. 2020b; Zheng et al. 2018b); Symptom Index of Dyspepsia (SID) (Ma et al. 2012).

### taVNS

We applied the same prescreening method as mentioned earlier and retrieved eight articles, reviewed abstracts, and full texts. Four papers matched the requirements and were included in the report (Table 3). Additionally, we included two studies that used percutaneous electrical nerve stimulation (PENS) for managing visceral and somatic pain. We extracted the following outcomes: Visual Analog Scale (VAS) (Shi et al. 2021); overall symptom scale (Wu et al. 2021); and dyspepsia symptom scale (Zhu et al. 2021).

### TEA/TEAS

After prescreening, we retrieved 22 articles, the abstracts and full texts were reviewed, and six papers matched the requirements and were included for reporting (Table 4). The following outcomes were extracted: visual analog pain score (VAS) (Hu et al. 2022; Huang et al. 2022; Xing et al. 2004); GERD questionnaire (GERDQ) (Hu et al. 2020; Zhang et al. 2021a).

## Mechanisms of action

Visceral hypersensitivity (VH) is a key factor that contributes to visceral pain in patients with GI disorders. The cell bodies of primary visceral afferents are located in the nodose ganglia (vagal afferents) and dorsal root ganglia (spinal afferents). Vagal sensory afferents are terminated in the NTS, whereas the central terminals of spinal visceral afferent neurons are organized segmentally. A growing body of literature suggests that visceral afferents convey noxious stimuli from the viscera under pathophysiological conditions (Gebhart 2000). The terminals of primary visceral afferents are located in the mucosa, muscles, and serosa. Visceral afferent nerve terminals respond to inflammatory mediators, chemical mediators, and mechanical stimuli. Previous studies have reported increased expression of the capsaicin receptor and transient receptor potential vanilloid type-1 receptor in nerve fibers of the colonic mucosa (Cheung et al. 2018; Wood 2007). Hence, afferent sensitization is an important factor contributing to visceral pain in patients with GI disorders.

Low-grade inflammation in the GI tract is associated with abdominal pain in various GI disorders, such as FD, IBD, IBS, and GERD. Multiple studies have investigated the link between low-grade inflammation and cytokines in GI disorders, providing evidence for their involvement in the pathogenesis of these conditions. Cytokines, both pro-inflammatory and anti-inflammatory, are small peptide proteins primarily secreted by immune cells. They exert their biological effects by interacting with specific cell surface receptors (Przemioslo and Ciclitira 1996) and play a crucial role in facilitating communication between cells, stimulating the proliferation of antigen-specific effector cells, and mediating local and systemic inflammation, including intestinal inflammation (Neuman 2007; Sanchez-Munoz et al. 2008). The interaction between cytokines and pain in GI disorders involves both peripheral and central mechanisms. Peripheral mechanisms involve the direct effects of cytokines on gut tissues and nerves, contributing to the sensation of pain. Previous studies in humans and animals with gastroparesis, IBS, FD, and IBD have reported increased levels of pro-inflammatory cytokines such as interleukin (IL)-1β, IL-6, IL-8, and tumor necrosis factor (TNF)-α in the blood and serum (Abell et al. 2021; de Souza and Fiocchi 2016; Dinan et al. 2006; Enck et al. 2017; Liebregts et al. 2007; van Heel et al. 2002). Elevated levels of these cytokines promote the inflammatory response within the GI tract, resulting in chronic inflammation. This chronic inflammation can lead to pain and discomfort, which are hallmark symptoms of these disorders (Akiho et al. 2010; Choghakhori et al. 2017; Friedrich et al. 2019; Mitselou et al. 2020; Neurath 2022; Strober et al. 2002). Other studies suggested that cytokines are associated with sensitization of the enteric nerves in the gut and heighten the responsiveness of these nerves to pain signals, leading to heightened pain perception in GI disorders (Barbara et al. 2004; Wang et al. 2022; Xia et al. 1999; Yoo and Mazmanian 2017). Central mechanisms involve processing pain signals in the brain and are influenced by both peripheral and psychological factors. Pain signals originating from the gut are transmitted to the CNS via well-defined ascending pathways, and cytokines can modulate the transmission and interpretation of these signals, contributing to the induction and maintenance of chronic pain (Alam and Chen 2023; Enck et al. 2017; Kawasaki et al. 2008). Given that GI disorders are considered bio-psychosocial disorders, psychological experiences such as stress, anxiety, and depression can interact with central mechanisms to influence pain perception. Cytokines can impact mood and psychological well-being, which, in turn, can affect the perception of pain in GI disorders (Khandaker et al. 2014; Strouse 2007). Furthermore, cytokines can alter normal GI motility, and these motility disturbances can be associated with abdominal discomfort and pain (Akiho et al. 2011; Ohama et al. 2008, 2007). Other studies have reported mucosal alterations and altered mucosal cytokine production in GI disorders (Enck et al. 2017; Ford and Talley 2011; Vanheel et al. 2014). Another clinical study demonstrated that increased levels of 5-HT in IBS patients contribute to abdominal pain (Cremon et al. 2011).

Moreover, numerous studies using transgenic animals, cholinergic agonists, and vagotomy support the role of the cholinergic anti-inflammatory pathway (CAP) in GI disorders (Bonaz 2007; Borovikova et al. 2000; Ghia et al. 2006; Goverse et al. 2016). It involves the activation of the parasympathetic nervous system and the release of acetylcholine, a neurotransmitter that binds to cholinergic receptors on immune cells (Pavlov et al. 2003; Tracey 2002). Dysregulation of the CAP can contribute to the chronic inflammation observed in the intestinal mucosa and regulate the synthesis of proinflammatory cytokines, including TNF- α and IL-1, thus contributing to the visceral pain in GI disorders. Therefore, low-grade inflammation may contribute to the pathogenesis of visceral pain in GI disorders by producing pro-inflammatory cytokines and dysregulating the CAP.

The gastrointestinal epithelium provides a barrier to the external environment, allowing the absorption of water and nutrients and limiting the permeation of luminal toxins and antigens through the mucosa. However, human and animal studies have reported increased intestinal permeability in GI disorders (Ahmad et al. 2017; Camilleri et al. 2012; Nojkov et al. 2020; Vanheel et al. 2014). Therefore, impaired intestinal barrier function and increased permeability may contribute to the pathophysiology of GI disorders and pain.

Mast cells have an immunoregulatory function at the mucosal border, and their overproduction or overactivation can contribute to the pathophysiology of GI disorders, including visceral pain (Ramsay et al. 2010). Increased numbers of mast cells have been reported in the ileum and mucosa of the colon, and overactivated mast cells release mediators, such as tryptase and histamine (Barbara et al. 2004, 2007; Hall et al. 2003; Reed et al. 2003; Weston et al. 1993). These mediators activate visceral sensory nerves and cause neuronal hyperexcitability, suggesting that mast cell activation leads to visceral hyperalgesia/allodynia and contributes to pain by affecting the sensorimotor function. Below, we discuss the mechanisms of action for each neuromodulation method.

### Mechanisms of acupuncture and EA for treating visceral pain

Acupuncture and EA exert their effects on visceral pain through peripheral, spinal, and supraspinal mechanisms (Zhang et al. 2014b). EA inhibits visceral pain through various peripheral chemicals, including neurotransmitters, neuropeptides, and cytokines. EA at ST36 significantly decreased visceral pain and colon 5-HT3 receptor levels (Chu et al. 2011), and EA at ST25 and ST37 significantly decreased the colorectal distension (CRD)-induced abdominal withdrawal reflex (AWR), number of mucosal mast cells, expression of corticotropin-releasing hormone (CRH) in the hypothalamus, and expression of Substance P and its receptor in the colon of rats with IBS (Ma et al. 2009; Wu et al. 2008). Several studies have demonstrated that acupuncture and EA reduce the expression of pro-inflammatory biomarkers, including myeloperoxidase (MPO), nitric oxide synthase (NOS), serum TNF-α, and colonic TNF-α mRNA, and increase the levels of anti-inflammatory cytokines, such as serum IL-10 (Goes et al. 2014; Kim et al. 2017; Tian et al. 2003). Another study reported that acupuncture inhibits epithelial cell apoptosis (Wu et al. 2004). EA modulates gastric motility by increasing the expression of nNOS in the antrum (Chen et al. 2016) and decreasing inflammation via expression of the tyrosine kinase receptor c-Kit in the gastric wall, and restoring the ICC network (Chen et al. 2013). These studies demonstrated that acupuncture and EA decrease numerous chemicals at peripheral sites to desensitize visceral afferents and improve visceral pain.

Acupuncture and EA may also attenuate visceral pain through the spinal and supraspinal mechanisms. Pre-EA at acupoint EX-B2 significantly inhibited intracolonic formalin-induced visceral pain. It decreases p38 phosphorylation and c-Fos expression in the spinal cord and colon, indicating that acupuncture and EA modulate spinal neuronal activity (Xu et al. 2010). In a rat model of IBS, EA at ST37 decreased visceral sensitivity to colorectal distension (CRD) and hypothalamic CRH to control synthesis (Wu et al. 2009). Moreover, the injection of the N-methyl-D-aspartate (NMDA) receptor antagonist D-2-amino-5-phosphonopentanoate (AP5) into the rostral ventromedial medulla (RVM) inhibited visceral pain (Sanoja et al. 2010). EA at ST36 and ST37 decrease c-Fos expression in the RVM (Qi and Li 2012), suggesting that acupuncture and EA may relieve visceral pain by inhibiting NMDAR activation in the RVM. Furthermore, acupuncture induces changes in the homeostatic afferent network, including the insula, ACC, and hypothalamus, suggesting that regulation of the CNS might be a specific mechanism of acupuncture (Zeng et al. 2012). Other studies have demonstrated that acupuncture and EA improve gastric hypersensitivity by regulating the vagal tone and sympathetic activity (Liu et al. 2012; Zhang et al. 2018b, 2020; Zhou et al. 2017).

The central mechanism of acupuncture and EA for pain in IBS has been extensively studied using multiple neuroimaging techniques such as functional magnetic resonance imaging (fMRI) and positron emission tomography (PET) (Ma et al. 2020; Xu et al. 2022a; Zhao et al. 2021). These studies have suggested that acupuncture and EA may have analgesic effects by modulating the default mode network and sensorimotor network (Zhao et al. 2021). Another recent study demonstrated that IBS patients have abnormal functional connections (FCs) in several brain areas, including the hippocampus, occipital gyrus, and cerebellum, and acupuncture treatment improved these FCs (Chu et al. 2012; Ma et al. 2020). Taken together, based on the studies presented here, we hypothesized that acupuncture and EA would alleviate visceral pain in GI disorders by regulating the peripheral, central, and endocrine systems. However, additional research could unravel the causal relationships between these systems.

### Mechanisms of taVNS for managing visceral pain

The vagus nerve exerts anti-inflammatory effects by activating both afferent and efferent fibers. Vagal afferents originating from the gut contain various receptors, including chemoreceptors, mechanoreceptors, thermoreceptors, and osmoreceptors. They can sense the functional state of the GI tract and activate afferent fibers (Goggins et al. 2022). Vagal efferent fibers originating from the brain innervate the GI tract. Peripheral stimuli are transmitted to the CNS via vagal afferent fibers ending in the NTS. The NTS sends this information to other brain regions, including the parabrachial nucleus, hypothalamus, and limbic system, including the amygdala, thalamus, hippocampus, cerebral cortex, insula, and prefrontal cortex (Bonaz et al. 2021; Cheng et al. 2020). A review reported that inflammation and cytokine imbalance are potential etiological factors for GI disorders and may be associated with visceral pain (Ford and Talley 2011). Both taVNS and needle-based VNS decrease TNF-α and IL-6 levels in IBS patients (Shi et al. 2021) and in a rodent model of TNBS-induced colitis (Jin et al. 2017), suggesting that the effect of VNS is mediated through the inhibition of pro-inflammatory cytokines.

Studies have shown that VNS activates CAP and exerts its anti-inflammatory effect through the release of acetylcholine. This pathway plays a critical role in controlling the inflammatory responses. Acetylcholine interacts with α7 nicotinic acetylcholine receptors (α7nAChRs) on macrophages, inhibiting the release of proinflammatory cytokines. Previous studies have suggested that VNS reduces intestinal inflammation and the production of serum TNF-α and IL-6 in animals and patients with IBS (Borovikova et al. 2000; Matteoli et al. 2014; Wang et al. 2003). Another study showed that taVNS decreased plasma 5-HT levels in IBS patients, which were positively correlated with visual analog scores (VAS) (Shi et al. 2021). These findings lead us to speculate that taVNS decreases inflammation by activating the cholinergic anti-inflammatory pathway and, in turn, alleviates visceral pain.

taVNS may ameliorate visceral pain by regulating the autonomic nervous system. Previous studies have reported that taVNS and VNS enhance and suppress parasympathetic and sympathetic nerve activity in healthy humans, respectively (Clancy et al. 2014; He et al. 2016). taVNS and VNS improve GI dysmotility by enhancing vagal activity and decreasing sympathetic activity in healthy volunteers and a rodent model of burn-induced gastric dysmotility (Frokjaer et al. 2016; Li et al. 2016). VNS decreases nociceptive behavior in animal models of VH by increasing vagal afferent excitability, suggesting an antinociceptive effect of VNS (Zurowski et al. 2012). Studies in animals and humans have demonstrated that auricular EA and taVNS enhance vagal tone and improve gastric hypersensitivity in a rodent model of FD (Zhou et al. 2017) and prevent the development of acid-induced esophageal hypersensitivity (Botha et al. 2015; Farmer et al. 2020; Zhou et al. 2017). These findings suggest that taVNS enhances vagal activity and improves colonic motility and visceral hypersensitivity, leading to reduced visceral pain.

Imaging techniques such as fMRI have been used for assessing the central mechanisms of taVNS for migraine. A clinical study in patients with migraine suggested that taVNS increase the connectivity between the motor thalamus and ACC/mPFC and decrease the connectivity between the occipital thalamus and postcentral gyrus (Zhang et al. 2021b). Other studies in patients with migraines demonstrated that taVNS increased FCs between the PAG and the middle cingulate cortex (MCC) (Cao et al. 2021) and between the LC with the right para-hippocampus and left amygdala (Zhang et al. 2019b), suggesting the effects on the pain modulation system. Based on the literature presented here, taVNS may have an analgesic effect on visceral pain in GI disorders through similar central mechanisms.

### Mechanisms of TEA for the management of visceral pain

TEA may share common mechanisms with acupuncture, EA, and taVNS in managing visceral pain in GI disorders. TEA treatment at ST36 improved stress-induced gastric slow-wave impairment by balancing sympathovagal activities (Zhang et al. 2015), and TEA at ST36 and PC6 improved gastric accommodation, gastric slow-wave damage, and dyspeptic symptoms in healthy volunteers (Huang et al. 2016). TEA improved nausea and gastric slow-wave abnormalities in patients with diabetic gastroparesis (Sarosiek et al. 2017). Another study has reported the role of TEA in the treatment of chronic functional constipation by increasing the frequency of spontaneous defecation and anorectal motility. These effects could be mediated by increased vagal activity and decreased sympathetic activity as measured by HRV (Zhang et al. 2014a). A recent human study demonstrated that TEA improved GI motility by increasing vagal activity and suppressing sympathetic activity, suggesting that TEA and taVNS share similar mechanisms.

TEA has been shown to reduce visceral pain in GI disorders, as evidenced by reduced VAS pain scores (Huang et al. 2022; Zhang et al. 2018a). The effects and mechanisms of EA on pain have been studied extensively, as discussed in the previous section. From our previous discussions and based on the studies reported here, we hypothesized that TEA shares the mechanism with EA and taVNS and inhibits pain through the peripheral, spinal, and supraspinal pathways with the involvement of a series of bioactive molecules, including opioids, serotonin, norepinephrine, cytokines, and signaling molecules.

## Discussion

In this review, we have introduced a few emerging noninvasive neuromodulation methods, summarized their mechanisms of action, and provided an overview of the effectiveness of these methods in treating visceral pain in GI disorders. In most of the reviewed studies, the acupuncture treatment protocol was applied every other day in the clinical setting. In contrast, the taVNS and TEAS treatment protocols were applied once or twice daily for up to 60 min for each treatment session. Although acupuncture and EA have been extensively studied for the management of pain in GI disorders, taVNS and TEA are relatively new, and these methods have mainly been studied in patients with IBS or FD. We have limited clinical trials for other conditions discussed in this review, such as gastroparesis, IBD, and NCCP. Based on the literature published to date, these methods are relatively safe, and to the best of our knowledge, severe side effects such as hospitalization have not been documented. Below, we summarize the three neuromodulation methods discussed previously.

**Table:** 

Stimulation method	Stimulation Location	Stimulation Frequency	Advantages	Disadvantages	Safety
Acupuncture/EA	Acupuncture points	1–100 Hz	Specific stimulation	Needle insertion, clinic cost	Overall safe, though bruising, fainting, and bleeding to the insertion site are reported in some studies.
taVNS	Auricular vagus nerve via surface electrodes	1–100 Hz	Self-administered, home-based, low cost	Daily placement of electrodes	Safe, some patients may have irritation from the electrodes
TEA/TEAS	Acupuncture points near the peripheral nerves	1–100 Hz	Self-administered, home-based, low cost	Daily placement of electrodes	Safe, some patients may experience irritation from the electrodes

In addition to the neuromodulation methods we have discussed, a recent noninvasive cervical vagus nerve stimulation method, known as “gammaCore” (electroCore; Basking Ridge, New Jersey, USA)” has received FDA approval for treating pain in patients with migraine and cluster headaches (Barbanti et al. 2015; Goadsby et al. 2014; Silberstein et al. 2016). This method stimulates myelinated sensory afferent fibers of the vagus nerve through the neck and has been used to treat drug-refractory gastroparesis (Paulon et al. 2017). Reviewing research data from clinical and animal studies leads us to propose that these noninvasive neuromodulation methods are promising tools for the treatment of pain in GI disorders, at least in theory. However, given the heterogeneous and multifactorial nature of these disorders, their chronicity, and the high placebo response, future studies are required to specify their efficacy more precisely. There is limited data on the long-term effects of these methods; therefore, long-term randomized controlled trials are needed to validate their chronic effects. Furthermore, optimization and curation of the methodology and study design are critically important.

Moreover, crosstalk between the gut and brain is essential for understanding the pathophysiology of pain in GI disorders, and how these two systems communicate is still in its early infancy. Peripheral pain stimuli are transmitted to the brain through anatomically and functionally distinct medial and lateral pain pathways. During chronic pain states, both pain pathways become dysfunctional. Research on animals and clinical data from human studies have proposed that abnormal brain oscillatory activity, such as theta, alpha, and beta, maybe a pathophysiological mechanism for pain in GI disorders. The role of theta oscillations in cognition and memory has been well-studied, and pain-specific theta band changes have been documented in GI disorders, although the data are minimal. Thus, brain oscillations could be a key factor in decoding pain sensations, and large-scale neuronal population recordings could be suitable for studying pain mechanisms. With clinical trials, further research is needed to explore the mechanism of action to better understand the therapeutic effect of noninvasive neuromodulation in managing visceral pain in GI disorders. Additionally, fMRI might be a good method for assessing central mechanisms involved in noninvasive neuromodulation for pain in GI disorders.

Based on the collective evidence derived from previous studies, noninvasive neuromodulation methods are emerging tools for managing visceral pain in the aforementioned disorders and related symptoms involving different segments of the gut. Given the heterogeneous nature of these diseases and their limited pathophysiological understanding, treatment options are minimal and controversial. Therefore, noninvasive neuromodulation is an attractive alternative for the treatment of pain in GI disorders.

### Supplementary Information

**Additional file 1.** Supplementary method.

## Data Availability

Not applicable.

## References

[CR1] Abell TL, Kedar A, Stocker A, Beatty K, McElmurray L, Hughes M, Rashed H, Kennedy W, Wendelschafer-Crabb G, Yang X, Fraig M, Gobejishvili L, Omer E, Miller E, Griswold M, Pinkston C (2021). Pathophysiology of gastroparesis syndromes includes anatomic and physiologic abnormalities. Dig Dis Sci.

[CR2] Afra P, Adamolekun B, Aydemir S, Watson GDR (2021). Evolution of the vagus nerve stimulation (VNS) therapy system technology for drug-resistant epilepsy. Front Med Technol.

[CR3] Ahmad R, Sorrell MF, Batra SK, Dhawan P, Singh AB (2017). Gut permeability and mucosal inflammation: bad, good or context dependent. Mucosal Immunol.

[CR4] Akiho H, Ihara E, Nakamura K (2010). Low-grade inflammation plays a pivotal role in gastrointestinal dysfunction in irritable bowel syndrome. World J Gastrointest Pathophysiol.

[CR5] Akiho H, Ihara E, Motomura Y, Nakamura K (2011). Cytokine-induced alterations of gastrointestinal motility in gastrointestinal disorders. World J Gastrointest Pathophysiol.

[CR6] Alam MJ, Chen JDZ (2023). Electrophysiology as a tool to decipher the network mechanism of visceral pain in functional gastrointestinal disorders. Diagnostics (Basel).

[CR7] Bahar RJ, Collins BS, Steinmetz B, Ament ME (2008). Double-blind placebo-controlled trial of amitriptyline for the treatment of irritable bowel syndrome in adolescents. J Pediatr.

[CR8] Bao CH, Zhao JM, Liu HR, Lu Y, Zhu YF, Shi Y, Weng ZJ, Feng H, Guan X, Li J, Chen WF, Wu LY, Jin XM, Dou CZ, Wu HG (2014). Randomized controlled trial: moxibustion and acupuncture for the treatment of Crohn’s disease. World J Gastroenterol.

[CR9] Bao C, Wu L, Wang D, Chen L, Jin X, Shi Y, Li G, Zhang J, Zeng X, Chen J, Liu H, Wu H (2022). Acupuncture improves the symptoms, intestinal microbiota, and inflammation of patients with mild to moderate Crohn’s disease: a randomized controlled trial. EClinicalMedicine.

[CR10] Barbanti P, Grazzi L, Egeo G, Padovan AM, Liebler E, Bussone G (2015). Non-invasive vagus nerve stimulation for acute treatment of high-frequency and chronic migraine: an open-label study. J Headache Pain.

[CR11] Barbara G, Stanghellini V, De Giorgio R, Cremon C, Cottrell GS, Santini D, Pasquinelli G, Morselli-Labate AM, Grady EF, Bunnett NW, Collins SM, Corinaldesi R (2004). Activated mast cells in proximity to colonic nerves correlate with abdominal pain in irritable bowel syndrome. Gastroenterology.

[CR12] Barbara G, Wang B, Stanghellini V, de Giorgio R, Cremon C, Di Nardo G, Trevisani M, Campi B, Geppetti P, Tonini M, Bunnett NW, Grundy D, Corinaldesi R (2007). Mast cell-dependent excitation of visceral-nociceptive sensory neurons in irritable bowel syndrome. Gastroenterology.

[CR13] Bari AA, Thum J, Babayan D, Lozano AM (2018). Current and expected advances in deep brain stimulation for movement disorders. Prog Neurol Surg.

[CR14] Bauer S, Baier H, Baumgartner C, Bohlmann K, Fauser S, Graf W, Hillenbrand B, Hirsch M, Last C, Lerche H, Mayer T, Schulze-Bonhage A, Steinhoff BJ, Weber Y, Hartlep A, Rosenow F, Hamer HM (2016). Transcutaneous vagus nerve stimulation (tVNS) for treatment of drug-resistant epilepsy: a randomized, double-blind clinical trial (cMPsE02). Brain Stimul.

[CR15] Baumgart DC, Carding SR (2007). Inflammatory bowel disease: cause and immunobiology. Lancet.

[CR16] Bergfeld IO, Dijkstra E, Graat I, de Koning P, van den Boom BJG, Arbab T, Vulink N, Denys D, Willuhn I, Mocking RJT (2021). Invasive and non-invasive neurostimulation for OCD. Curr Top Behav Neurosci.

[CR17] Bonaz B (2007). The cholinergic anti-inflammatory pathway and the gastrointestinal tract. Gastroenterology.

[CR18] Bonaz B, Picq C, Sinniger V, Mayol JF, Clarencon D (2013). Vagus nerve stimulation: from epilepsy to the cholinergic anti-inflammatory pathway. Neurogastroenterol Motil.

[CR19] Bonaz B, Sinniger V, Pellissier S (2021). Therapeutic potential of vagus nerve stimulation for inflammatory bowel diseases. Front Neurosci.

[CR20] Borovikova LV, Ivanova S, Zhang M, Yang H, Botchkina GI, Watkins LR, Wang H, Abumrad N, Eaton JW, Tracey KJ (2000). Vagus nerve stimulation attenuates the systemic inflammatory response to endotoxin. Nature.

[CR21] Botha C, Farmer AD, Nilsson M, Brock C, Gavrila AD, Drewes AM, Knowles CH, Aziz Q (2015). Preliminary report: modulation of parasympathetic nervous system tone influences oesophageal pain hypersensitivity. Gut.

[CR22] Camilleri M, Lasch K, Zhou W (2012). Irritable bowel syndrome: methods, mechanisms, and pathophysiology. The confluence of increased permeability, inflammation, and pain in irritable bowel syndrome. Am J Physiol Gastrointest Liver Physiol.

[CR23] Cao J, Zhang Y, Li H, Yan Z, Liu X, Hou X, Chen W, Hodges S, Kong J, Liu B (2021). Different modulation effects of 1 Hz and 20 Hz transcutaneous auricular vagus nerve stimulation on the functional connectivity of the periaqueductal gray in patients with migraine. J Transl Med.

[CR24] Chen Y, Xu JJ, Liu S, Hou XH (2013). Electroacupuncture at ST36 ameliorates gastric emptying and rescues networks of interstitial cells of Cajal in the stomach of diabetic rats. PLoS One.

[CR25] Chen H, Zhu W, Lu J, Fan J, Sun L, Feng X, Liu H, Zhang Z, Wang Y (2016). The effects of auricular electro-acupuncture on ameliorating the dysfunction of interstitial cells of cajal networks and nNOSmRNA expression in antrum of STZ-induced diabetic rats. PLoS One.

[CR26] Chen J, Tu Q, Miao S, Zhou Z, Hu S (2020). Transcutaneous electrical acupoint stimulation for preventing postoperative nausea and vomiting after general anesthesia: a meta-analysis of randomized controlled trials. Int J Surg.

[CR27] Chen CH, Tsai TC, Wu YJ, Hsu KS (2023). Gastric vagal afferent signaling to the basolateral amygdala mediates anxiety-like behaviors in experimental colitis mice. JCI Insight.

[CR28] Cheng RS, Pomeranz B (1979). Electroacupuncture analgesia could be mediated by at least two pain-relieving mechanisms; endorphin and non-endorphin systems. Life Sci.

[CR29] Cheng J, Shen H, Chowdhury R, Abdi T, Selaru F, Chen JDZ (2020). Potential of electrical neuromodulation for inflammatory bowel disease. Inflamm Bowel Dis.

[CR30] Cheung CKY, Lan LL, Kyaw M, Mak ADP, Chan A, Chan Y, Wu JCY. Up-regulation of transient receptor potential vanilloid (TRPV) and down-regulation of brain-derived neurotrophic factor (BDNF) expression in patients with functional dyspepsia (FD). Neurogastroenterol Motil. 2018;30(2). 10.1111/nmo.13176. 10.1111/nmo.13176 28782273

[CR31] Choghakhori R, Abbasnezhad A, Hasanvand A, Amani R (2017). Inflammatory cytokines and oxidative stress biomarkers in irritable bowel syndrome: association with digestive symptoms and quality of life. Cytokine.

[CR32] Chu D, Cheng P, Xiong H, Zhang J, Liu S, Hou X (2011). Electroacupuncture at ST-36 relieves visceral hypersensitivity and decreases 5-HT(3) receptor level in the colon in chronic visceral hypersensitivity rats. Int J Colorectal Dis.

[CR33] Chu WC, Wu JC, Yew DT, Zhang L, Shi L, Yeung DK, Wang D, Tong RK, Chan Y, Lao L, Leung PC, Berman BM, Sung JJ (2012). Does acupuncture therapy alter activation of neural pathway for pain perception in irritable bowel syndrome?: a comparative study of true and sham acupuncture using functional magnetic resonance imaging. J Neurogastroenterol Motil.

[CR34] Clancy JA, Mary DA, Witte KK, Greenwood JP, Deuchars SA, Deuchars J (2014). Non-invasive vagus nerve stimulation in healthy humans reduces sympathetic nerve activity. Brain Stimul.

[CR35] Cooper CM, Farrand AQ, Andresen MC, Beaumont E (2021). Vagus nerve stimulation activates nucleus of solitary tract neurons via supramedullary pathways. J Physiol.

[CR36] Cremon C, Carini G, Wang B, Vasina V, Cogliandro RF, De Giorgio R, Stanghellini V, Grundy D, Tonini M, De Ponti F, Corinaldesi R, Barbara G (2011). Intestinal serotonin release, sensory neuron activation, and abdominal pain in irritable bowel syndrome. Am J Gastroenterol.

[CR37] Crowell MD, Jones MP, Harris LA, Dineen TN, Schettler VA, Olden KW (2004). Antidepressants in the treatment of irritable bowel syndrome and visceral pain syndromes. Curr Opin Investig Drugs.

[CR38] de Souza HS, Fiocchi C (2016). Immunopathogenesis of IBD: current state of the art. Nat Rev Gastroenterol Hepatol.

[CR39] Diener HC, Kronfeld K, Boewing G, Lungenhausen M, Maier C, Molsberger A, Tegenthoff M, Trampisch HJ, Zenz M, Meinert R, Group GMS (2006). Efficacy of acupuncture for the prophylaxis of migraine: a multicentre randomised controlled clinical trial. Lancet Neurol.

[CR40] Dinan TG, Quigley EM, Ahmed SM, Scully P, O’Brien S, O’Mahony L, O’Mahony S, Shanahan F, Keeling PW (2006). Hypothalamic-pituitary-gut axis dysregulation in irritable bowel syndrome: plasma cytokines as a potential biomarker?. Gastroenterology.

[CR41] Enck P, Azpiroz F, Boeckxstaens G, Elsenbruch S, Feinle-Bisset C, Holtmann G, Lackner JM, Ronkainen J, Schemann M, Stengel A, Tack J, Zipfel S, Talley NJ (2017). Functional dyspepsia. Nat Rev Dis Primers.

[CR42] Farmer AD, Albusoda A, Amarasinghe G, Ruffle JK, Fitzke HE, Idrees R, Fried R, Brock C, Aziz Q (2020). Transcutaneous vagus nerve stimulation prevents the development of, and reverses, established oesophageal pain hypersensitivity. Aliment Pharmacol Ther.

[CR43] Fass R, Navarro-Rodriguez T (2008). Noncardiac chest pain. J Clin Gastroenterol.

[CR44] Fischer R, Ventura-Bort C, Hamm A, Weymar M (2018). Transcutaneous vagus nerve stimulation (tVNS) enhances conflict-triggered adjustment of cognitive control. Cogn Affect Behav Neurosci.

[CR45] Ford AC, Talley NJ (2011). Mucosal inflammation as a potential etiological factor in irritable bowel syndrome: a systematic review. J Gastroenterol.

[CR46] Francis RP, Johnson MI (2011). The characteristics of acupuncture-like transcutaneous electrical nerve stimulation (acupuncture-like TENS): a literature review. Acupunct Electrother Res.

[CR47] Friedrich M, Pohin M, Powrie F (2019). Cytokine networks in the pathophysiology of inflammatory bowel disease. Immunity.

[CR48] Frokjaer JB, Bergmann S, Brock C, Madzak A, Farmer AD, Ellrich J, Drewes AM (2016). Modulation of vagal tone enhances gastroduodenal motility and reduces somatic pain sensitivity. Neurogastroenterol Motil.

[CR49] Gebhart GF (2000). Pathobiology of visceral pain: molecular mechanisms and therapeutic implications IV. Visceral afferent contributions to the pathobiology of visceral pain. Am J Physiol Gastrointest Liver Physiol.

[CR50] Ghia JE, Blennerhassett P, Kumar-Ondiveeran H, Verdu EF, Collins SM (2006). The vagus nerve: a tonic inhibitory influence associated with inflammatory bowel disease in a murine model. Gastroenterology.

[CR51] Goadsby PJ, Grosberg BM, Mauskop A, Cady R, Simmons KA (2014). Effect of noninvasive vagus nerve stimulation on acute migraine: an open-label pilot study. Cephalalgia.

[CR52] Goes AC, Pinto FM, Fernandes GC, Barbosa JS, Correia ES, Ribeiro RA, Guimaraes SB, Lima Junior RC, Brito GA, Rodrigues LV (2014). Electroacupuncture ameliorates experimental colitis induced by TNBS through activation of interleukin-10 and inhibition of iNOS in mice. Acta Cir Bras.

[CR53] Goggins E, Mitani S, Tanaka S (2022). Clinical perspectives on vagus nerve stimulation: present and future. Clin Sci (lond).

[CR54] Gorard DA, Libby GW, Farthing MJ (1994). Influence of antidepressants on whole gut and orocaecal transit times in health and irritable bowel syndrome. Aliment Pharmacol Ther.

[CR55] Goverse G, Stakenborg M, Matteoli G (2016). The intestinal cholinergic anti-inflammatory pathway. J Physiol.

[CR56] Grover M, Drossman DA (2008). Psychotropic agents in functional gastrointestinal disorders. Curr Opin Pharmacol.

[CR57] Hall W, Buckley M, Crotty P, O’Morain CA (2003). Gastric mucosal mast cells are increased in Helicobacter pylori-negative functional dyspepsia. Clin Gastroenterol Hepatol.

[CR58] He B, Lu Z, He W, Huang B, Jiang H (2016). Autonomic modulation by electrical stimulation of the parasympathetic nervous system: an emerging intervention for cardiovascular diseases. Cardiovasc Ther.

[CR59] Hein E, Nowak M, Kiess O, Biermann T, Bayerlein K, Kornhuber J, Kraus T (2013). Auricular transcutaneous electrical nerve stimulation in depressed patients: a randomized controlled pilot study. J Neural Transm (vienna).

[CR60] Hu Y, Zhang B, Shi X, Ning B, Shi J, Zeng X, Liu F, Chen JD, Xie WF (2020). Ameliorating effects and autonomic mechanisms of transcutaneous electrical acustimulation in patients with gastroesophageal reflux disease. Neuromodulation.

[CR61] Hu P, Sun K, Li H, Qi X, Gong J, Zhang Y, Xu L, Lin M, Fan Y, Chen JDZ (2022). Transcutaneous electrical acustimulation improved the quality of life in patients with diarrhea-irritable bowel syndrome. Neuromodulation.

[CR62] Huang Z, Zhang N, Xu F, Yin J, Dai N, Chen JD (2016). Ameliorating effect of transcutaneous electroacupuncture on impaired gastric accommodation induced by cold meal in healthy subjects. J Gastroenterol Hepatol.

[CR63] Huang Z, Lin Z, Lin C, Chu H, Zheng X, Chen B, Du L, Chen JDZ, Dai N (2022). Transcutaneous electrical acustimulation improves irritable bowel syndrome with constipation by accelerating colon transit and reducing rectal sensation using autonomic mechanisms. Am J Gastroenterol.

[CR64] Jerlock M, Welin C, Rosengren A, Gaston-Johansson F (2007). Pain characteristics in patients with unexplained chest pain and patients with ischemic heart disease. Eur J Cardiovasc Nurs.

[CR65] Ji T, Li X, Lin L, Jiang L, Wang M, Zhou X, Zhang R, Chen J (2014). An alternative to current therapies of functional dyspepsia: self-administrated transcutaneous electroacupuncture improves dyspeptic symptoms. Evid Based Complement Alternat Med.

[CR66] Jiang J, Liu H, Wang Z, Tian H, Wang S, Yang J, Ren J (2021). Electroacupuncture could balance the gut microbiota and improve the learning and memory abilities of Alzheimer’s disease animal model. PLoS One.

[CR67] Jin Y, Zhao Q, Zhou K, Jing X, Yu X, Fang J, Liu Z, Zhu B (2015). Acupuncture for functional dyspepsia: a single blinded, randomized, controlled trial. Evid Based Complement Alternat Med.

[CR68] Jin H, Guo J, Liu J, Lyu B, Foreman RD, Yin J, Shi Z, Chen JDZ (2017). Anti-inflammatory effects and mechanisms of vagal nerve stimulation combined with electroacupuncture in a rodent model of TNBS-induced colitis. Am J Physiol Gastrointest Liver Physiol.

[CR69] Kawasaki Y, Zhang L, Cheng JK, Ji RR (2008). Cytokine mechanisms of central sensitization: distinct and overlapping role of interleukin-1beta, interleukin-6, and tumor necrosis factor-alpha in regulating synaptic and neuronal activity in the superficial spinal cord. J Neurosci.

[CR70] Khandaker GM, Pearson RM, Zammit S, Lewis G, Jones PB (2014). Association of serum interleukin 6 and C-reactive protein in childhood with depression and psychosis in young adult life: a population-based longitudinal study. JAMA Psychiat.

[CR71] Kim BJ, Kuo B (2019). Gastroparesis and functional dyspepsia: a blurring distinction of pathophysiology and treatment. J Neurogastroenterol Motil.

[CR72] Kim DH, Ryu Y, Hahm DH, Sohn BY, Shim I, Kwon OS, Chang S, Gwak YS, Kim MS, Kim JH, Lee BH, Jang EY, Zhao R, Chung JM, Yang CH, Kim HY (2017). Acupuncture points can be identified as cutaneous neurogenic inflammatory spots. Sci Rep.

[CR73] Ko SJ, Kuo B, Kim SK, Lee H, Kim J, Han G, Kim J, Kim SY, Jang S, Son J, Kim M, Lee H, Yeo I, Joo KR, Park JW (2016). Individualized acupuncture for symptom relief in functional dyspepsia: a randomized controlled trial. J Altern Complement Med.

[CR74] Kovacic K, Hainsworth K, Sood M, Chelimsky G, Unteutsch R, Nugent M, Simpson P, Miranda A (2017). Neurostimulation for abdominal pain-related functional gastrointestinal disorders in adolescents: a randomised, double-blind, sham-controlled trial. Lancet Gastroenterol Hepatol.

[CR75] Krames ES, Hunter Peckham P, Rezai A, Aboelsaad F. Chapter 1 - what is neuromodulation? In: Krames ES, Peckham PH, Rezai AR, editors. Neuromodulation. Academic Press; 2009. pp. 3–8. 10.1016/B978-0-12-374248-3.00002-1.

[CR76] Krasaelap A, Sood MR, Li BUK, Unteutsch R, Yan K, Nugent M, Simpson P, Kovacic K (2020). Efficacy of auricular neurostimulation in adolescents with irritable bowel syndrome in a randomized, double-blind trial. Clin Gastroenterol Hepatol.

[CR77] Lebel AA (2008). Pharmacology. J Pediatr Gastroenterol Nutr.

[CR78] Lee B, Kwon OJ, Kim JH, Kang JW, Kim TH, Lee S, Kim J, Kim AR, Jung SY, Park HJ, Choi SM (2022). Saam acupuncture for treating functional dyspepsia: a feasibility randomized controlled trial. Evid Based Complement Alternat Med.

[CR79] Lehtimaki J, Hyvarinen P, Ylikoski M, Bergholm M, Makela JP, Aarnisalo A, Pirvola U, Makitie A, Ylikoski J (2013). Transcutaneous vagus nerve stimulation in tinnitus: a pilot study. Acta Otolaryngol.

[CR80] Li GM, Huang CF, Zhang XZ, Xie H, Cheng HY, Tang YS, Li ZG (2015). The short-term effects of acupuncture on patients with diabetic gastroparesis: a randomised crossover study. Acupunct Med.

[CR81] Li H, Yin J, Zhang Z, Winston JH, Shi XZ, Chen JD (2016). Auricular vagal nerve stimulation ameliorates burn-induced gastric dysmotility via sympathetic-COX-2 pathways in rats. Neurogastroenterol Motil.

[CR82] Li WJ, Gao C, An LX, Ji YW, Xue FS, Du Y (2021). Perioperative transcutaneous electrical acupoint stimulation for improving postoperative gastrointestinal function: a randomized controlled trial. J Integr Med.

[CR83] Liebregts T, Adam B, Bredack C, Roth A, Heinzel S, Lester S, Downie-Doyle S, Smith E, Drew P, Talley NJ, Holtmann G (2007). Immune activation in patients with irritable bowel syndrome. Gastroenterology.

[CR84] Linde K, Streng A, Jurgens S, Hoppe A, Brinkhaus B, Witt C, Wagenpfeil S, Pfaffenrath V, Hammes MG, Weidenhammer W, Willich SN, Melchart D (2005). Acupuncture for patients with migraine: a randomized controlled trial. JAMA.

[CR85] Liu S, Peng S, Hou X, Ke M, Chen JD (2008). Transcutaneous electroacupuncture improves dyspeptic symptoms and increases high frequency heart rate variability in patients with functional dyspepsia. Neurogastroenterol Motil.

[CR86] Liu J, Huang H, Xu X, Chen JD (2012). Effects and possible mechanisms of acupuncture at ST36 on upper and lower abdominal symptoms induced by rectal distension in healthy volunteers. Am J Physiol Regul Integr Comp Physiol.

[CR87] Liu CH, Yang MH, Zhang GZ, Wang XX, Li B, Li M, Woelfer M, Walter M, Wang L (2020). Neural networks and the anti-inflammatory effect of transcutaneous auricular vagus nerve stimulation in depression. J Neuroinflammation.

[CR88] Liu HH, Zhang GS, Liu HJ, Li DD, Liu M, Chang XR, Liu ML (2020). Acupuncture therapy with point selection based on syndrome differentiation along the meridians for functional dyspepsia: a randomized controlled trial. J Acupunct Tuina Sci.

[CR89] Lopes LT, Patrone LG, Li KY, Imber AN, Graham CD, Gargaglioni LH, Putnam RW (2016). Anatomical and functional connections between the locus coeruleus and the nucleus tractus solitarius in neonatal rats. Neuroscience.

[CR90] Lowe C, Aiken A, Day AG, Depew W, Vanner SJ (2017). Sham acupuncture is as efficacious as true acupuncture for the treatment of IBS: a randomized placebo controlled trial. Neurogastroenterol Motil.

[CR91] Ma XP, Tan LY, Yang Y, Wu HG, Jiang B, Liu HR, Yang L (2009). Effect of electro-acupuncture on substance P, its receptor and corticotropin-releasing hormone in rats with irritable bowel syndrome. World J Gastroenterol.

[CR92] Ma TT, Yu SY, Li Y, Liang FR, Tian XP, Zheng H, Yan J, Sun GJ, Chang XR, Zhao L, Wu X, Zeng F (2012). Randomised clinical trial: an assessment of acupuncture on specific meridian or specific acupoint vs. sham acupuncture for treating functional dyspepsia. Aliment Pharmacol Ther.

[CR93] Ma K, Liu Y, Shao W, Sun J, Li J, Fang X, Li J, Wang Z, Zhang D (2020). Brain functional interaction of acupuncture effects in diarrhea-dominant irritable bowel syndrome. Front Neurosci.

[CR94] Madisch A, Andresen V, Enck P, Labenz J, Frieling T, Schemann M (2018). The diagnosis and treatment of functional dyspepsia. Dtsch Arztebl Int.

[CR95] Mahmood A, Veluswamy SK, Hombali A, Mullick ANM, Solomon JM (2019). Effect of transcutaneous electrical nerve stimulation on spasticity in adults with stroke: a systematic review and meta-analysis. Arch Phys Med Rehabil.

[CR96] Matteoli G, Gomez-Pinilla PJ, Nemethova A, Di Giovangiulio M, Cailotto C, van Bree SH, Michel K, Tracey KJ, Schemann M, Boesmans W, Vanden Berghe P, Boeckxstaens GE (2014). A distinct vagal anti-inflammatory pathway modulates intestinal muscularis resident macrophages independent of the spleen. Gut.

[CR97] Mayer EA, Ryu HJ, Bhatt RR (2023). The neurobiology of irritable bowel syndrome. Mol Psychiatry.

[CR98] Meng GJ (2019). Acupuncture treatment for depressive symptom in diarrhea-predominant irritable bowel syndrome: a randomized controlled study. J Acupunct Tuina Sci.

[CR99] Mion F, Pellissier S, Garros A, Damon H, Roman S, Bonaz B (2020). Transcutaneous auricular vagus nerve stimulation for the treatment of irritable bowel syndrome: a pilot, open-label study. Bioelectron Med.

[CR100] Mitselou A, Grammeniatis V, Varouktsi A, Papadatos SS, Katsanos K, Galani V (2020). Proinflammatory cytokines in irritable bowel syndrome: a comparison with inflammatory bowel disease. Intest Res.

[CR101] Mudipalli RS, Remes-Troche JM, Andersen L, Rao SS (2007). Functional chest pain: esophageal or overlapping functional disorder. J Clin Gastroenterol.

[CR102] Napadow V, Edwards RR, Cahalan CM, Mensing G, Greenbaum S, Valovska A, Li A, Kim J, Maeda Y, Park K, Wasan AD (2012). Evoked pain analgesia in chronic pelvic pain patients using respiratory-gated auricular vagal afferent nerve stimulation. Pain Med.

[CR103] Neuman MG (2007). Immune dysfunction in inflammatory bowel disease. Transl Res.

[CR104] Neurath MF (2022). Targeting cytokines in inflammatory bowel disease. Sci Transl Med.

[CR105] Nojkov B, Zhou SY, Dolan RD, Davis EM, Appelman HD, Guo X, Jackson K, Sturm MB, Wang TD, Owyang C, Liu JJ, Chey WD (2020). Evidence of duodenal epithelial barrier impairment and increased pyroptosis in patients with functional dyspepsia on confocal laser endomicroscopy and “ex vivo” mucosa analysis. Am J Gastroenterol.

[CR106] Ohama T, Hori M, Momotani E, Iwakura Y, Guo F, Kishi H, Kobayashi S, Ozaki H (2007). Intestinal inflammation downregulates smooth muscle CPI-17 through induction of TNF-alpha and causes motility disorders. Am J Physiol Gastrointest Liver Physiol.

[CR107] Ohama T, Hori M, Fujisawa M, Kiyosue M, Hashimoto M, Ikenoue Y, Jinno Y, Miwa H, Matsumoto T, Murata T, Ozaki H (2008). Downregulation of CPI-17 contributes to dysfunctional motility in chronic intestinal inflammation model mice and ulcerative colitis patients. J Gastroenterol.

[CR108] Page MJ, McKenzie JE, Bossuyt PM, Boutron I, Hoffmann TC, Mulrow CD, Shamseer L, Tetzlaff JM, Akl EA, Brennan SE, Chou R, Glanville J, Grimshaw JM, Hrobjartsson A, Lalu MM, Li T, Loder EW, Mayo-Wilson E, McDonald S, McGuinness LA, Stewart LA, Thomas J, Tricco AC, Welch VA, Whiting P, Moher D (2021). The PRISMA 2020 statement: an updated guideline for reporting systematic reviews. Syst Rev.

[CR109] Park YC, Kang W, Choi SM, Son CG (2009). Evaluation of manual acupuncture at classical and nondefined points for treatment of functional dyspepsia: a randomized-controlled trial. J Altern Complement Med.

[CR110] Parkman HP (2015). Idiopathic gastroparesis. Gastroenterol Clin North Am.

[CR111] Parseliunas A, Paskauskas S, Kubiliute E, Vaitekunas J, Venskutonis D (2021). Transcutaneous electric nerve stimulation reduces acute postoperative pain and analgesic use after open inguinal hernia surgery: a randomized, double-blind, placebo-controlled trial. J Pain.

[CR112] Paulon E, Nastou D, Jaboli F, Marin J, Liebler E, Epstein O (2017). Proof of concept: short-term non-invasive cervical vagus nerve stimulation in patients with drug-refractory gastroparesis. Frontline Gastroenterol.

[CR113] Pavlov VA, Wang H, Czura CJ, Friedman SG, Tracey KJ (2003). The cholinergic anti-inflammatory pathway: a missing link in neuroimmunomodulation. Mol Med.

[CR114] Pei LX, Geng H, Guo J, Yang GH, Wang L, Shen RR, Xia SY, Ding M, Feng H, Lu J, Li J, Liu L, Shu YY, Fang XD, Wu XL, Wang XX, Weng SJ, Ju L, Chen X, Shen H, Sun JH (2020). Effect of acupuncture in patients with irritable bowel syndrome: a randomized controlled trial. Mayo Clin Proc.

[CR115] Pomeranz B, Warma N (1988). Electroacupuncture suppression of a nociceptive reflex is potentiated by two repeated electroacupuncture treatments: the first opioid effect potentiates a second non-opioid effect. Brain Res.

[CR116] Przemioslo RT, Ciclitira PJ (1996). Cytokines and gastrointestinal disease mechanisms. Baillieres Clin Gastroenterol.

[CR117] Qi DB, Li WM (2012). Effects of electroacupuncture on expression of c-fos protein and N-methyl-D-aspartate receptor 1 in the rostral ventromedial medulla of rats with chronic visceral hyperalgesia. Zhong Xi Yi Jie He Xue Bao.

[CR118] Qi LY, Yang JW, Yan SY, Tu JF, She YF, Li Y, Chi LL, Wu BQ, Liu CZ (2022). Acupuncture for the treatment of diarrhea-predominant irritable bowel syndrome: a pilot randomized clinical trial. JAMA Netw Open.

[CR119] Rafiei R, Ataie M, Ramezani MA, Etemadi A, Ataei B, Nikyar H, Abdoli S (2014). A new acupuncture method for management of irritable bowel syndrome: a randomized double blind clinical trial. J Res Med Sci.

[CR120] Ramsay DB, Stephen S, Borum M, Voltaggio L, Doman DB (2010). Mast cells in gastrointestinal disease. Gastroenterol Hepatol (N Y).

[CR121] Redgrave J, Day D, Leung H, Laud PJ, Ali A, Lindert R, Majid A (2018). Safety and tolerability of transcutaneous vagus nerve stimulation in humans; a systematic review. Brain Stimul.

[CR122] Reed DE, Barajas-Lopez C, Cottrell G, Velazquez-Rocha S, Dery O, Grady EF, Bunnett NW, Vanner SJ (2003). Mast cell tryptase and proteinase-activated receptor 2 induce hyperexcitability of guinea-pig submucosal neurons. J Physiol.

[CR123] Sahn B, Pascuma K, Kohn N, Tracey KJ, Markowitz JF (2023). Transcutaneous auricular vagus nerve stimulation attenuates inflammatory bowel disease in children: a proof-of-concept clinical trial. Bioelectron Med.

[CR124] Sanchez-Munoz F, Dominguez-Lopez A, Yamamoto-Furusho JK (2008). Role of cytokines in inflammatory bowel disease. World J Gastroenterol.

[CR125] Sanoja R, Tortorici V, Fernandez C, Price TJ, Cervero F (2010). Role of RVM neurons in capsaicin-evoked visceral nociception and referred hyperalgesia. Eur J Pain.

[CR126] Sarosiek I, Song G, Sun Y, Sandoval H, Sands S, Chen J, McCallum RW (2017). Central and peripheral effects of transcutaneous acupuncture treatment for nausea in patients with diabetic gastroparesis. J Neurogastroenterol Motil.

[CR127] See MC, Birnbaum AH, Schechter CB, Goldenberg MM, Benkov KJ (2001). Double-blind, placebo-controlled trial of famotidine in children with abdominal pain and dyspepsia: global and quantitative assessment. Dig Dis Sci.

[CR128] Shen JH, Ye YM, Li SS (2022). Acupuncture for diarrhea-predominant irritable bowel syndrome: a randomized control study. World J Acupunct Moxibustion.

[CR129] Shi X, Hu Y, Zhang B, Li W, Chen JD, Liu F (2021). Ameliorating effects and mechanisms of transcutaneous auricular vagal nerve stimulation on abdominal pain and constipation. JCI Insight.

[CR130] Shi X, Zhao L, Luo H, Deng H, Wang X, Ren G, Zhang L, Tao Q, Liang S, Liu N, Huang X, Zhang X, Yang X, Sun J, Qin W, Kang X, Han Y, Pan Y, Fan D. Transcutaneous auricular vagal nerve stimulation is effective for the treatment of functional dyspepsia: a multicenter, randomized controlled study. Am J Gastroenterol. 2023. 10.14309/ajg.0000000000002548. 10.14309/ajg.0000000000002548 37787432

[CR131] Silberstein SD, Mechtler LL, Kudrow DB, Calhoun AH, McClure C, Saper JR, Liebler EJ, Rubenstein Engel E, Tepper SJ, Group ACTS (2016). Non-invasive vagus nerve stimulation for the ACute Treatment of cluster headache: findings from the randomized, double-blind, sham-controlled ACT1 study. Headache.

[CR132] Song G, Sun Y, Bashashati M, Quezada A, Sigaroodi S, Sarosiek I, Chen JDZ, McCallum RW (2018). Efficacy of needleless transcutaneous electroacupuncture in synchronization with breathing for symptomatic idiopathic gastroparesis: a blinded and controlled acute treatment trial. Neurogastroenterol Motil.

[CR133] Song G, Fiocchi C, Achkar JP (2019). Acupuncture in inflammatory bowel disease. Inflamm Bowel Dis.

[CR134] Steidel K, Krause K, Menzler K, Strzelczyk A, Immisch I, Fuest S, Gorny I, Mross P, Hakel L, Schmidt L, Timmermann L, Rosenow F, Bauer S, Knake S (2021). Transcutaneous auricular vagus nerve stimulation influences gastric motility: a randomized, double-blind trial in healthy individuals. Brain Stimul.

[CR135] Strober W, Fuss IJ, Blumberg RS (2002). The immunology of mucosal models of inflammation. Annu Rev Immunol.

[CR136] Strober W, Fuss I, Mannon P (2007). The fundamental basis of inflammatory bowel disease. J Clin Invest.

[CR137] Strouse TB (2007). The relationship between cytokines and pain/depression: a review and current status. Curr Pain Headache Rep.

[CR138] Tian L, Huang YX, Tian M, Gao W, Chang Q (2003). Downregulation of electroacupuncture at ST36 on TNF-alpha in rats with ulcerative colitis. World J Gastroenterol.

[CR139] Tracey KJ (2002). The inflammatory reflex. Nature.

[CR140] Tu Q, Gan J, Shi J, Yu H, He S, Zhang J (2019). Effect of transcutaneous electrical acupoint stimulation on postoperative analgesia after ureteroscopic lithotripsy: a randomized controlled trial. Urolithiasis.

[CR141] van Heel DA, Udalova IA, De Silva AP, McGovern DP, Kinouchi Y, Hull J, Lench NJ, Cardon LR, Carey AH, Jewell DP, Kwiatkowski D (2002). Inflammatory bowel disease is associated with a TNF polymorphism that affects an interaction between the OCT1 and NF(-kappa)B transcription factors. Hum Mol Genet.

[CR142] Vanheel H, Vicario M, Vanuytsel T, Van Oudenhove L, Martinez C, Keita AV, Pardon N, Santos J, Soderholm JD, Tack J, Farre R (2014). Impaired duodenal mucosal integrity and low-grade inflammation in functional dyspepsia. Gut.

[CR143] Wang H, Yu M, Ochani M, Amella CA, Tanovic M, Susarla S, Li JH, Wang H, Yang H, Ulloa L, Al-Abed Y, Czura CJ, Tracey KJ (2003). Nicotinic acetylcholine receptor alpha7 subunit is an essential regulator of inflammation. Nature.

[CR144] Wang H, Foong JPP, Harris NL, Bornstein JC (2022). Enteric neuroimmune interactions coordinate intestinal responses in health and disease. Mucosal Immunol.

[CR145] Wang H, Xiang Y, Wang C, Wang Y, Chen S, Ding L, Liu Q, Wang X, Zhao K, Jia J, Chen Y (2023). Effects of transcutaneous electrical acupoint stimulation on upper-limb impairment after stroke: a randomized, controlled, single-blind trial. Clin Rehabil.

[CR146] Weaver FM, Follett K, Stern M, Hur K, Harris C, Marks WJ, Rothlind J, Sagher O, Reda D, Moy CS, Pahwa R, Burchiel K, Hogarth P, Lai EC, Duda JE, Holloway K, Samii A, Horn S, Bronstein J, Stoner G, Heemskerk J, Huang GD, Group CSPS (2009). Bilateral deep brain stimulation vs best medical therapy for patients with advanced Parkinson disease: a randomized controlled trial. JAMA.

[CR147] Weston AP, Biddle WL, Bhatia PS, Miner PB (1993). Terminal ileal mucosal mast cells in irritable bowel syndrome. Dig Dis Sci.

[CR148] White A, Ernst E (2004). A brief history of acupuncture. Rheumatology (Oxford).

[CR149] Whitfield KL, Shulman RJ (2009). Treatment options for functional gastrointestinal disorders: from empiric to complementary approaches. Pediatr Ann.

[CR150] Wood JD (2007). Neuropathophysiology of functional gastrointestinal disorders. World J Gastroenterol.

[CR151] Wu HG, Gong X, Yao LQ, Zhang W, Shi Y, Liu HR, Gong YJ, Zhou LB, Zhu Y (2004). Mechanisms of acupuncture and moxibustion in regulation of epithelial cell apoptosis in rat ulcerative colitis. World J Gastroenterol.

[CR152] Wu HG, Jiang B, Zhou EH, Shi Z, Shi DR, Cui YH, Kou ST, Liu HR (2008). Regulatory mechanism of electroacupuncture in irritable bowel syndrome: preventing MC activation and decreasing SP VIP secretion. Dig Dis Sci.

[CR153] Wu HG, Liu HR, Zhang ZA, Zhou EH, Wang XM, Jiang B, Shi Z, Zhou CL, Qi L, Ma XP (2009). Electro-acupuncture relieves visceral sensitivity and decreases hypothalamic corticotropin-releasing hormone levels in a rat model of irritable bowel syndrome. Neurosci Lett.

[CR154] Wu X, Zheng CH, Xu XH, Ding P, Xiong F, Tian M, Wang Y, Dong HX, Zhang MM, Wang W, Xu SB, Xie MJ, Huang GY (2017). Electroacupuncture for functional constipation: a multicenter, randomized, control trial. Evid Based Complement Alternat Med.

[CR155] Wu D, Wang Y, Zhang JL, Lian HH, Chen LQ, Peng T, Rong PJ, Hou LW (2021). Transcutaneous auricular vagus nerve stimulation for functional dyspepsia: a randomized controlled trial. World J Acupunct Moxibustion.

[CR156] Wu L, Dong Y, Zhu C, Chen Y (2023). Effect and mechanism of acupuncture on Alzheimer’s disease: a review. Front Aging Neurosci.

[CR157] Xia Y, Hu HZ, Liu S, Ren J, Zafirov DH, Wood JD (1999). IL-1beta and IL-6 excite neurons and suppress nicotinic and noradrenergic neurotransmission in guinea pig enteric nervous system. J Clin Invest.

[CR158] Xiao Y, Xu F, Lin L, Chen JDZ (2022). Transcutaneous electrical acustimulation improves constipation by enhancing rectal sensation in patients with functional constipation and lack of rectal sensation. Clin Transl Gastroenterol.

[CR159] Xing JH, Larive B, Mekhail N, Soffer E (2004). Transcutaneous electrical acustimulation can reduce visceral perception in patients with the irritable bowel syndrome: a pilot study. Altern Ther Health Med.

[CR160] Xu KD, Liang T, Wang K, Tian DA (2010). Effect of pre-electroacupuncture on p38 and c-Fos expression in the spinal dorsal horn of rats suffering from visceral pain. Chin Med J (Engl).

[CR161] Xu J, Xie H, Liu L, Shen Z, Yang L, Wei W, Guo X, Liang F, Yu S, Yang J (2022). Brain mechanism of acupuncture treatment of chronic pain: an individual-level positron emission tomography study. Front Neurol.

[CR162] Xu XH, Zhang MM, Wu X, Zheng CH, Huang GY (2022). The effect of electroacupuncture treatment with different intensities for functional diarrhea: a randomized controlled trial. Evid Based Complement Alternat Med.

[CR163] Xuefen W, Ping L, Li L, Xiaoli C, Yue Z (2020). A clinical randomized controlled trial of acupuncture treatment of gastroparesis using different acupoints. Pain Res Manag.

[CR164] Yang J, Huang L, Liu S, Wu W, Tian W, Zheng Z, Lv Z, Ji F, Zheng M (2020). Effect of electroacupuncture on postoperative gastrointestinal recovery in patients undergoing thoracoscopic surgery: a feasibility study. Med Sci Monit.

[CR165] Yap JYY, Keatch C, Lambert E, Woods W, Stoddart PR, Kameneva T (2020). Critical review of transcutaneous vagus nerve stimulation: challenges for translation to clinical practice. Front Neurosci.

[CR166] Yin J, Chen JD (2010). Gastrointestinal motility disorders and acupuncture. Auton Neurosci.

[CR167] Yoo BB, Mazmanian SK (2017). The enteric network: interactions between the immune and nervous systems of the gut. Immunity.

[CR168] Yu ZJ, Weller RA, Sandidge K, Weller EB (2008). Vagus nerve stimulation: can it be used in adolescents or children with treatment-resistant depression?. Curr Psychiatry Rep.

[CR169] Zeng F, Qin W, Ma T, Sun J, Tang Y, Yuan K, Li Y, Liu J, Liu X, Song W, Lan L, Liu M, Yu S, Gao X, Tian J, Liang F (2012). Influence of acupuncture treatment on cerebral activity in functional dyspepsia patients and its relationship with efficacy. Am J Gastroenterol.

[CR170] Zhang N, Huang Z, Xu F, Xu Y, Chen J, Yin J, Lin L, Chen JD (2014). Transcutaneous neuromodulation at posterior tibial nerve and ST36 for chronic constipation. Evid Based Complement Alternat Med.

[CR171] Zhang R, Lao L, Ren K, Berman BM (2014). Mechanisms of acupuncture-electroacupuncture on persistent pain. Anesthesiology.

[CR172] Zhang N, Song G, Chen J, Xu F, Yin J, Wu Q, Lin L, Chen JD (2015). Ameliorating effects and autonomic mechanisms of needle-less transcutaneous electrical stimulation at ST36 on stress-induced impairment in gastric slow waves. J Gastroenterol Hepatol.

[CR173] Zhang B, Xu F, Hu P, Zhang M, Tong K, Ma G, Xu Y, Zhu L, Chen JDZ (2018). Needleless transcutaneous electrical acustimulation: a pilot study evaluating improvement in post-operative recovery. Am J Gastroenterol.

[CR174] Zhang S, Li S, Liu Y, Ye F, Yin J, Foreman RD, Wang D, Chen JDZ (2018). Electroacupuncture via chronically implanted electrodes improves gastric dysmotility mediated by autonomic-cholinergic mechanisms in a rodent model of functional dyspepsia. Neurogastroenterol Motil.

[CR175] Zhang X, Ding M, Feng H (2019). Acupuncture with Du’s heat-reinforcing method for diarrhea-predominant irritable bowel syndrome: a randomized controlled trial. J Acupunct Tuina Sci.

[CR176] Zhang Y, Liu J, Li H, Yan Z, Liu X, Cao J, Park J, Wilson G, Liu B, Kong J (2019). Transcutaneous auricular vagus nerve stimulation at 1 Hz modulates locus coeruleus activity and resting state functional connectivity in patients with migraine: an fMRI study. Neuroimage Clin.

[CR177] Zhang S, Liu Y, Li S, Ye F, Foreman RD, Chen JDZ (2020). Effects of electroacupuncture on stress-induced gastric dysrhythmia and mechanisms involving autonomic and central nervous systems in functional dyspepsia. Am J Physiol Regul Integr Comp Physiol.

[CR178] Zhang B, Hu Y, Shi X, Li W, Zeng X, Liu F, Chen JDZ, Xie WF (2021). Integrative effects and vagal mechanisms of transcutaneous electrical acustimulation on gastroesophageal motility in patients with gastroesophageal reflux disease. Am J Gastroenterol.

[CR179] Zhang Y, Huang Y, Li H, Yan Z, Zhang Y, Liu X, Hou X, Chen W, Tu Y, Hodges S, Chen H, Liu B, Kong J (2021). Transcutaneous auricular vagus nerve stimulation (taVNS) for migraine: an fMRI study. Reg Anesth Pain Med.

[CR180] Zhang Y, Lu T, Meng Y, Maisiyiti A, Dong Y, Li S, Chen Y, Yin J, Chen JDZ (2021). Auricular vagal nerve stimulation improves constipation by enhancing colon motility via the central-vagal efferent pathway in opioid-induced constipated rats. Neuromodulation.

[CR181] Zhao ZQ (2008). Neural mechanism underlying acupuncture analgesia. Prog Neurobiol.

[CR182] Zhao T, Pei L, Ning H, Guo J, Song Y, Zhou J, Chen L, Sun J, Mi Z (2021). Networks are associated with acupuncture treatment in patients with diarrhea-predominant irritable bowel syndrome: a resting-state imaging study. Front Hum Neurosci.

[CR183] Zheng H, Li Y, Zhang W, Zeng F, Zhou SY, Zheng HB, Zhu WZ, Jing XH, Rong PJ, Tang CZ, Wang FC, Liu ZB, Wang SJ, Zhou MQ, Liu ZS, Zhu B (2016). Electroacupuncture for patients with diarrhea-predominant irritable bowel syndrome or functional diarrhea: a randomized controlled trial. Medicine.

[CR184] Zheng H, Liu ZS, Zhang W, Chen M, Zhong F, Jing XH, Rong PJ, Zhu WZ, Wang FC, Liu ZB, Tang CZ, Wang SJ, Zhou MQ, Li Y, Zhu B (2018). Acupuncture for patients with chronic functional constipation: a randomized controlled trial. Neurogastroenterol Motil.

[CR185] Zheng H, Xu J, Sun X, Zeng F, Li Y, Wu X, Li J, Zhao L, Chang XR, Liu M, Gong B, Li XZ, Liang FR (2018). Electroacupuncture for patients with refractory functional dyspepsia: a randomized controlled trial. Neurogastroenterol Motil.

[CR186] Zhou J, Li S, Wang Y, Lei Y, Foreman RD, Yin J, Chen JD (2017). Effects and mechanisms of auricular electroacupuncture on gastric hypersensitivity in a rodent model of functional dyspepsia. PLoS One.

[CR187] Zhou D, Hu B, He S, Li X, Gong H, Li F, Wang Q (2018). Transcutaneous electrical acupoint stimulation accelerates the recovery of gastrointestinal function after cesarean section: a randomized controlled trial. Evid Based Complement Alternat Med.

[CR188] Zhu Y, Xu F, Lu D, Rong P, Cheng J, Li M, Gong Y, Sun C, Wei W, Lin L, Chen JDZ (2021). Transcutaneous auricular vagal nerve stimulation improves functional dyspepsia by enhancing vagal efferent activity. Am J Physiol Gastrointest Liver Physiol.

[CR189] Zurowski D, Nowak L, Wordliczek J, Dobrogowski J, Thor PJ (2012). Effects of vagus nerve stimulation in visceral pain model. Folia Med Cracov.

