# Supplementary material for: Non-invasive neuromodulation: an emerging intervention for visceral pain in gastrointestinal disorders
Source: Bioelectron Med. 2023 Nov 22;9:27. doi: 10.1186/s42234-023-00130-5 (PMC10664460; doi:10.1186/s42234-023-00130-5)
Supplement: Supplementary file 1 — Additional file 1. Supplementary method. [file 42234_2023_130_MOESM1_ESM.docx]

Supplementary method

Title: Non‑invasive neuromodulation: an emerging intervention for visceral pain in gastrointestinal

disorders

Md Jahangir Alam., Ph.D. and Jiande DZ Chen., Ph.D.

Division of Gastroenterology and Hepatology, Department of Internal Medicine, University of Michigan, Ann Arbor, Michigan 48109

Correspondence should be addressed to Md Jahangir Alam., Ph.D. ([mdalam@med.umich.edu](mailto:mdalam@med.umich.edu)) and Jiande D. Z. Chen, Ph.D. ([cjiande@med.umich.edu](mailto:cjiande@med.umich.edu))

Supplementary method

1. Acupuncture and electroacupuncture

Search by condition keywords: ['Functional Dyspepsia', 'Dyspepsia', 'Noncardiac chest pain', 'Inflammatory bowel disease', 'IBD', 'Irritable bowel syndrome', 'IBS', 'Abdominal pain', 'visceral', 'gastrointestinal pain', 'sensitivity', 'Gastroparesis', 'delayed gastric emptying', 'gastric motility', 'gastric accommodation', 'Gastroesophageal Reflux Disease', 'constipation', 'Diarrhea'].

Dropping keywords: ['pig', 'rabbit', 'dog', 'retracted', 'cancer', 'rat', 'mice', 'moxibustion', 'cat', 'auricular', 'vagus', 'vagal', 'Systematic review', 'review', 'meta-analysis', 'meta analysis', 'sacral nerve stimulation', 'spinal cord', 'withdrawn', 'probiotic', 'combined', 'moxibuxtion', 'commentary', 'mouse', 'Cognitive Behavioral Therapy', ‘Cognitive-behavioral therapy', 'paroxetine', 'rodent', 'stroke', 'goats', 'animal'].

Final keywords: ['Acupuncture', 'Electroacupuncture'].

1. Transcutaneous auricular vagus nerve stimulation (taVNS) and auricular vagus nerve stimulation (aVNS)

Search by condition keywords: ['Functional Dyspepsia', 'Dyspepsia', 'Noncardiac chest pain', 'Inflammatory bowel disease', 'IBD', 'Irritable bowel syndrome', 'IBS', 'Abdominal pain', 'visceral', 'gastrointestinal pain', 'sensitivity', 'Gastroparesis', 'delayed gastric emptying', 'gastric motility', 'gastric accommodation', 'Gastroesophageal Reflux Disease', 'constipation', 'Diarrhea'].

Dropping keywords: ['pig', 'rabbit', 'dog', 'retracted', 'cancer', 'rat', 'mice', 'moxibustion', 'cat', 'Electroacupuncture', 'Systematic review', 'review', 'meta-analysis', 'meta analysis', 'sacral', 'spinal cord', 'withdrawn', 'probiotic', 'combined', 'moxibuxtion', 'commentary', 'mouse', 'Cognitive Behavioral Therapy', 'Cognitive-behavioral therapy', 'paroxetine', 'rodent', 'stroke', 'goats', 'animal', 'acupuncture'].

Final keywords: ['Auricular vagus nerve stimulation', 'Transcutaneous Auricular Vagus Nerve Stimulation', 'Auricular Vagal Nerve Stimulation', 'Transcutaneous Auricular Vagal Nerve Stimulation', 'Vagus Nerve Stimulation', 'Vagal Nerve Stimulation', 'aVNS', 'taVNS'].

1. Transcutaneous electrical stimulation (TEA) and Transcutaneous electrical acustimulation (TEAS)

Search by condition keywords: ['Functional Dyspepsia', 'Dyspepsia', 'Noncardiac chest pain', 'Inflammatory bowel disease', 'IBD', 'Irritable bowel syndrome', 'IBS', 'Abdominal pain', 'visceral', 'gastrointestinal pain', 'sensitivity', 'Gastroparesis', 'delayed gastric emptying', 'gastric motility', 'gastric accommodation', 'Gastroesophageal Reflux Disease', 'constipation', 'Diarrhea'].

Dropping keywords: ['pig', 'rabbit', 'dog', 'retracted', 'cancer', 'rat', 'mice', 'moxibustion', 'cat', 'auricular', 'Systematic review', 'review', 'meta-analysis', 'meta analysis', 'sacral', 'spinal cord', 'withdrawn', 'probiotic', 'combined', 'moxibuxtion', 'commentary', 'mouse', 'Cognitive Behavioral Therapy', 'Cognitive-behavioral therapy', 'paroxetine', 'rodent', 'stroke', 'goats', 'animal']

Final keywords: ['Transcutaneous electrical stimulation', 'Transcutaneous electrical acustimulation', 'Transcutaneous electrical acu-stimulation', 'stimulation', 'TEA', 'TEAS', 'transcutaneous electroacupuncture'].
